# CD62E- and ROS-Responsive ETS Improves Cartilage Repair by Inhibiting Endothelial Cell Activation through OPA1-Mediated Mitochondrial Homeostasis

**DOI:** 10.34133/bmr.0006

**Published:** 2024-03-04

**Authors:** Pengcheng Tu, Yalan Pan, Lining Wang, Bin Li, Xiaoxian Sun, Zhongqing Liang, Mengmin Liu, Zitong Zhao, Chengjie Wu, Jianwei Wang, Zhifang Wang, Yu Song, Yafeng Zhang, Yong Ma, Yang Guo

**Affiliations:** ^1^ Affiliated Hospital of Nanjing University of Chinese Medicine, Nanjing 210029, P.R. China.; ^2^ Laboratory of New Techniques of Restoration and Reconstruction of Orthopedics and Traumatology, Nanjing University of Chinese Medicine, Nanjing 210023, P.R. China.; ^3^ School of Chinese Medicine, School of Integrated Chinese and Western Medicine, Nanjing University of Chinese Medicine, Nanjing 210023, P.R. China.; ^4^ Key Laboratory of Acupuncture and Medicine Research of Ministry of Education, Nanjing University of Chinese Medicine, Nanjing 210023, China.; ^5^ School of Acupuncture and Tuina, School of Health and Rehabilitation, Nanjing University of Chinese Medicine, Nanjing 210046, Jiangsu, China.; ^6^ Jiangsu CM Clinical Innovation Center of Degenerative Bone & Joint Disease, Wuxi TCM Hospital Affiliated to Nanjing University of Chinese Medicine, Wuxi 214072, P.R. China.; ^7^ Zhangjiagang TCM Hospital Affiliated to Nanjing University of Chinese Medicine, Zhangjiagang 215600, P.R. China.; ^8^ Zhangjiagang First People’s Hospital Affiliated to Soochow University, Zhangjiagang 215638, P.R. China.

## Abstract

**Background:** In the environment of cartilage injury, the activation of vascular endothelial cell (VEC), marked with excessive CD62E and reactive oxygen species (ROS), can affect the formation of hyaluronic cartilage. Therefore, we developed a CD62E- and ROS-responsive drug delivery system using E-selectin binding peptide, Thioketal, and silk fibroin (ETS) to achieve targeted delivery and controlled release of Clematis triterpenoid saponins (CS) against activated VEC, and thus promote cartilage regeneration. **Methods:** We prepared and characterized ETS/CS and verified their CD62E- and ROS-responsive properties in vitro. We investigated the effect and underlying mechanism of ETS/CS on inhibiting VEC activation and promoting chondrogenic differentiation of bone marrow stromal cells (BMSCs). We also analyzed the effect of ETS/CS on suppressing the activated VEC-macrophage inflammatory cascade in vitro. Additionally, we constructed a rat knee cartilage defect model and administered ETS/CS combined with BMSC-containing hydrogels. We detected the cartilage differentiation, the level of VEC activation and macrophage in the new tissue, and synovial tissue. **Results:** ETS/CS was able to interact with VEC and inhibit VEC activation through the carried CS. Coculture experiments verified ETS/CS promoted chondrogenic differentiation of BMSCs by inhibiting the activated VEC-induced inflammatory cascade of macrophages via OPA1-mediated mitochondrial homeostasis. In the rat knee cartilage defect model, ETS/CS reduced VEC activation, migration, angiogenesis in new tissues, inhibited macrophage infiltration and inflammation, promoted chondrogenic differentiation of BMSCs in the defective areas. **Conclusions:** CD62E- and ROS-responsive ETS/CS promoted cartilage repair by inhibiting VEC activation and macrophage inflammation and promoting BMSC chondrogenesis. Therefore, it is a promising therapeutic strategy to promote articular cartilage repair.

## Introduction

Articular cartilage injury is difficult to repair and easily progresses to degenerative diseases of the knee joints [1]. The clinical treatment of this injury is still dominated by nonsteroidal anti-inflammatory drugs, which have no repair effect and elicit noticeable side effects on the cardiovascular system [2]. However, regenerated tissues treated using microfracture and other surgeries mainly consist of scars and fibrocartilage, which have poor long-term efficacy and are prone to degenerative changes [3]. Currently, cartilage tissue engineering (CTE) is considered an ideal method for achieving cartilage repair. In recent years, the in situ repair method using bone marrow stromal cells (BMSCs) combined with biological scaffolds and growth factors has become a research trend as it can improve the success rate of cartilage repair [4,5].

Articular cartilage is a clear, avascular tissue. However, after cartilage injury, the absence of a vascular barrier allows vascular endothelial cells (VECs) from the subchondral bone and joint cavity to easily activate and invade the engineered cartilage, leading to the recruitment of inflammatory cells. Inflammatory cells, such as macrophages, secrete a large number of cytokines [6,7], resulting in gradual fibrosis and endochondral osteogenesis of the newly formed hyaluronic cartilage, thus affecting the long-term efficacy of CTE [8–10]. Therefore, inhibiting VECs during CTE is of great significance in promoting the formation of high-quality hyaluronic cartilage during cartilage injury repair. After a cartilage injury, the local VECs can be activated by local inflammation, hypoxia, and other factors [11–13]. The phenotype and function of activated VECs change, with the up-regulation of E-selectin, one of the adhesion molecules, and enhanced ability to recruit inflammatory macrophages [13]. Studies have shown that various intracellular signaling pathways, including nuclear factor κB (NF-κB), mitogen-activated kinase kinase 1 (MEK1), and mitogen-activated protein kinase (MAPK), regulate VEC activation [14–16]. Recent studies have also indicated that intracellular second messengers, such as reactive oxygen species (ROS) and Ca^2+^, which are regulated by mitochondria, play a role in mediating VEC activation, proliferation, migration, and gene expression under various angiogenic stimuli [17,18]. Opa1 (Optic atrophy protein 1), located in the inner membrane of mitochondria, mediates mitochondrial inner fusion and is critical for promoting the stability of the respiratory chain complex, regulating ROS production and mitochondrial Ca^2+^ influx, and maintaining cytoplasmic homeostasis [19,20]. However, the exact mechanism of opa1 in improving the cartilage repair environment by mediating mitochondrial homeostasis of VEC activation remains to be verified.

In recent years, there has been a growing body of evidence supporting the use of bioactive components found in traditional Chinese medicines to promote tissue regeneration and repair as exogenous drugs [21,22]. One such bioactive compound is Clematis triterpenoid saponins (CS), derived from Clematidis Radix, a traditional Chinese medicine known for its protective effects on articular cartilage [23,24]. Studies have shown that CS has antiangiogenic effects [25,26], which may contribute to its protective effect on articular cartilage. It has also been found to improve mitochondrial oxidative stress damage and inhibit inflammation progression. Therefore, it is speculated that CS may inhibit the VEC activation-cartilage degeneration process by regulating OPA1-mitochondrial homeostasis. However, the low bioavailability and poor stability of CS in physiological environments may limit its efficacy, making it necessary to explore new dosage forms. Fibrin, a natural polymer, has good biocompatibility and morphological plasticity and is widely used in CTE [27]. Studies have shown that silk fibroin (SF) scaffolds loaded with CS can promote cartilage regeneration in a rabbit knee cartilage defect model [28]. SF, as a kind of biomaterial with good biocompatibility, degradability and modifiability, has been widely used in CTE. While simple SF-based drug delivery systems lack drug control release properties, stimulus-responsive materials combined with VEC-targeting characteristics offer possibilities for designing systems to precisely regulate drug release in space and time. E-selectin binding peptide (ESBP) is a ligand of E-selectin (CD62E), which enables the local enrichment of VEC-targeting nanoparticles through CD62E-mediated adhesion and endocytosis [29]. Thioketal (TK) is a ROS-responsive material, as ROS can trigger chain fracture of the TK polymer, resulting in responsive cracking to achieve controlled drug release [30].

Herein, we propose to modify SF with ESBP and TK to achieve CD62E-targeted adhesion and ROS-responsive drug release properties. Combined with the hydrogel packaging of the cartilage matrix network structure, we can achieve effective accumulation and release of CS in VECs and promote the reconstruction of tissue-engineered cartilage by inhibiting VEC activation. Therefore, in this study, ETS/CS with CD62E and ROS dual-responsiveness characteristics were prepared and characterized. In vitro experiments were conducted to elucidate the effect and mechanism of ETS/CS in inhibiting VEC activation by up-regulating OPA1-mediated mitochondrial homeostasis. Additionally, we investigated the potential of the ETS/CS system in promoting the chondrogenic differentiation of BMSCs and their interaction with VECs. The role of OPA1, a protein involved in mitochondrial homeostasis, was up-regulated by ETS/CS treatment, leading to enhanced mitochondrial function and reduced VEC activation. This finding suggests that ETS/CS can not only inhibit VEC activation but also support the chondrogenic differentiation of BMSCs. To further validate the therapeutic potential of our CD62E and ROS-responsive ETS/CS system, we conducted animal experiments. The ETS/CS hydrogels were implanted in cartilage injury models, and the results demonstrated their ability to inhibit VEC activation and promote high-quality repair of the injured cartilage. This indicates that the ETS/CS system has great potential for contributing to the development of effective strategies for cartilage tissue regeneration.

**Fig. 1.**
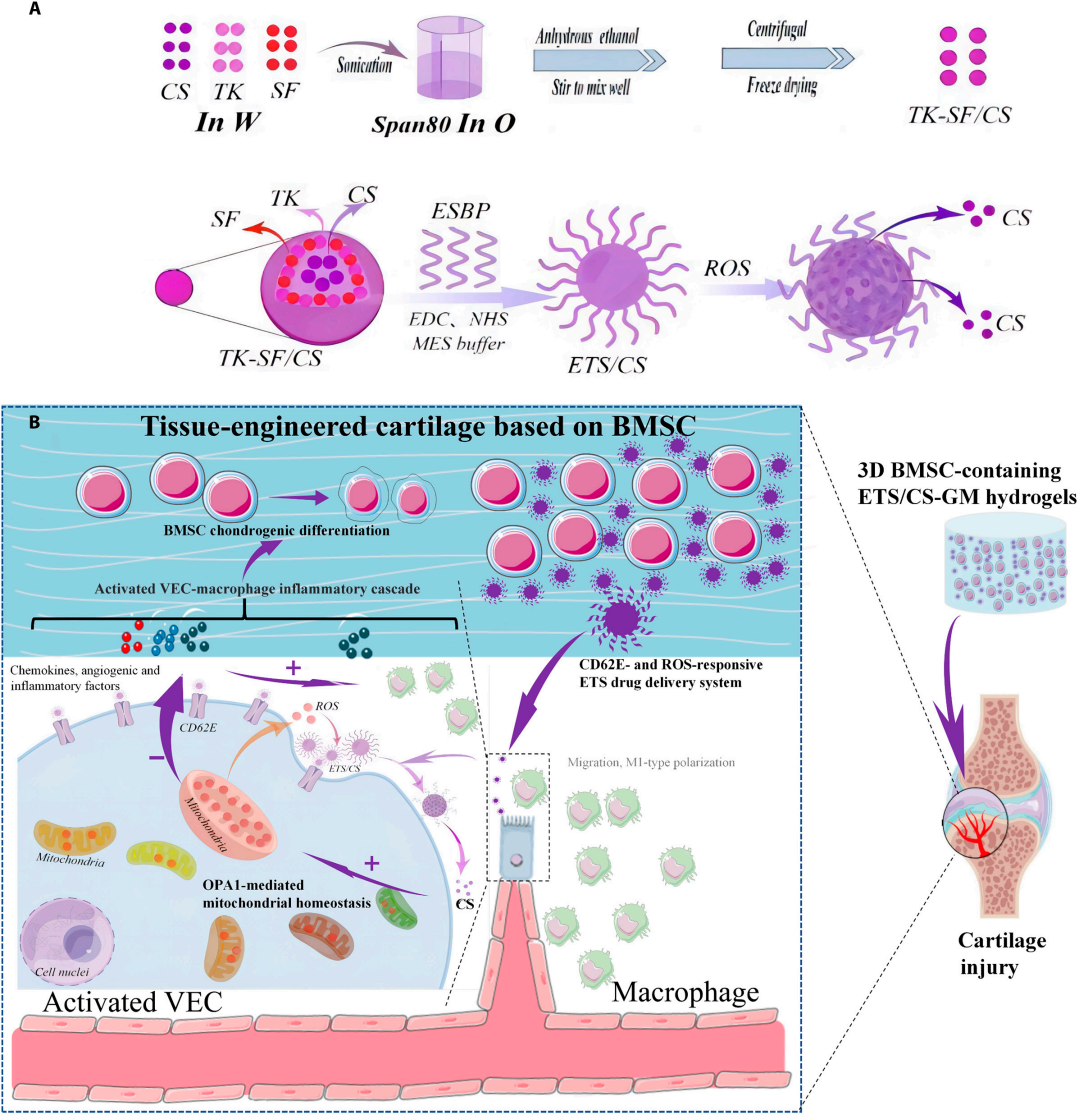
Schematic illustration of CD62E- and ROS-responsive ETS drug delivery system, its function and underlying mechanisms. (A) CD62E- and ROS-responsive ETS drug delivery system compromised ESBP (a ligand of E-selectin), TK (a ROS-responsive material), SF, and chondroprotective drug CS. (B) After 3D BMSC-containing ETS/CS-GM hydrogel was implanted into cartilage defect, ETS/CS targets CD62E+ activated VEC, releases CS in response to excessively high ROS level, and CS improves cartilage repair by inhibiting endothelial cell activation through OPA1-mediated mitochondrial homeostasis.

In summary, our study introduces a novel approach to modify SF by incorporating ESBP and TK. The resulting ETS/CS system exhibits CD62E-targeted adhesion, ROS-responsive drug release properties, and the ability to inhibit VEC activation (Fig. 1). Our findings highlight the importance of OPA1-mediated mitochondrial homeostasis in this process. Furthermore, the ETS/CS system shows promising results in promoting chondrogenic differentiation of BMSCs and facilitating cartilage repair. This research contributes to the field of tissue engineering and provides valuable insights for the development of advanced therapies for cartilage injuries.

## Materials and Methods

### Preparation of the CD62E and ROS-responsive ETS drug delivery system

#### Synthesis of TS

SF (125 mg) was dissolved in 5 ml of ultrapure water. Next, 25 mg of TK and 50 mg of CS were dissolved in SF solution (2.5%) as the aqueous phase. In parallel, 300 μl of Span80 was dispersed in 30 ml of soybean oil and stirred evenly in a 50 °C water bath (200 rpm, 5 min) as the oil phase. The aqueous phase was then added dropwise to the oil phase to prepare an emulsion using ultrasound (260 W, amplitude transformer Φ = 6 mm, 25 kHz, 5 s on/5 s off, 5 min). Subsequently, 5 ml of anhydrous ethanol was added to a 50 °C water bath for 2 h with magnetic stirring. The resulting mixture was then subjected to centrifugation after passing through a 40-μm sieve to collect the precipitate. The precipitate was dispersed in anhydrous ethanol to remove residual soybean oil, washed in ultrapure water, and subjected to centrifugation. Finally, the precipitate was lyophilized to obtain TS/CS. Similarly, SF/CS and TS without CS were prepared using the same method and stored at −20 °C until use.

#### Synthesis of ETS

The TS/CS or TS microspheres were initially dispersed in a 2 g/l NaOH solution and stirred magnetically at a speed of 200 rpm/min for 1 min. Subsequently, the microspheres were dispersed in a buffer solution (pH 6) containing 50 mM MES (2-(N-morpholino) ethanesulfonic acid), 25 mM NHS (N-hydroxysuccinimide), and 50 mM EDC (1-(3-dimethylaminopropyl)-3-ethylcarbodiimide hydrochloride). This mixture was stirred for 30 min in an ice bath to activate the carboxyl groups. After activation, the microspheres were placed in ESBP solution (0.5 mg/ml) and stirred at 25 °C for 1 h. Then, they were transferred to a 1% glycinate solution and stirred at room temperature for 5 min to quench the reaction. Finally, the resulting precipitate was collected, washed with ultrapure water, and subjected to lyophilization to obtain ETS/CS and ETS. To prepare fluorescein isothiocyanate (FITC)-ETS, FITC-labeled ESBP (obtained from Kingsley, Shanghai, China) was used while ensuring protection from light.

### Characterization of the ETS drug delivery system

The scanning electron microscope (SEM) images of ETS were acquired utilizing a HITACHI Regulus 8100 electron microscope. To determine the particle size distribution and zeta potential of the microspheres, a Zetasizer Nano ZS instrument was employed. Fourier transform infrared (FTIR) spectra were recorded using an IRTracer-100 spectrometer. Additionally, the microsphere powder was pressed into sheets for x-ray photoelectron spectroscopy (XPS) analysis, which was conducted using a Thermo Scientific K-Alpha system.

### CS loading efficiency and release tests

#### CS loading in the ETS

ETS/CS was added to a methanol, which was fully dispersed via ultrasound. The CS concentration in the supernatant was determined using the vanillin-perchloric acid colorimetric method [31], and oleanolic acid was used as a control. The encapsulation and loading rates were calculated using the following equations:

$$\begin{array}{c} \text{Encapsulation efficiency}\;\left(100\%\right)\;=\\ \;\text{Loaded CS}/\text{Total CS}\;\times \;100\% \end{array}$$

Loading efficiency=Loaded CS/ETS×100%

#### Controlled release characteristics of ETS/CS

ETS/CS (10 mg) was added to 5 ml of phosphate-buffered saline (PBS) and incubated at 37 °C without light. The supernatant was taken at 1, 2, 4, 8, 10, 12, and 14 d, replacing the supernatant taken with the same amount of fresh PBS solution. Similarly, ETS/CS (10 mg) was also placed in 5 ml of PBS containing H_2_O_2_ (100 μM) and incubated at 37 °C without light. The supernatant was collected after 1, 2, 3, 4, 12, and 16 h, replacing the supernatant taken with the same amount of fresh PBS solution. A blank ETS leaching solution was used as the control. The vanillin-perchloric acid colorimetric method was used to determine the CS content and calculate the cumulative release rate.

### Cell culture

A rat VEC line (RAOEC) was purchased from the BeNa Culture Collection (Beijing, China) and cultured in supplemented Dulbecco’s modified Eagle medium (DMEM) (containing 10% FBS [Gibco, USA] and 1% penicillin-streptomycin [KeyGEN, Jiangsu]) at 37 °C in an atmosphere of 5% CO_2_ [32]. Rat peritoneal macrophages (PMCs) were extracted via peritoneal lavage and cultured in supplemented DMEM at 37 °C and 5% CO_2_ [33]. Rat BMSCs were isolated using the whole bone marrow adherent method and cultured with supplemented DMEM at 37 °C and 5% CO_2_ [34]. BMSCs from the third passage were used in this study. For the molecular mechanism investigation, tumor necrosis factor-α (TNF-α) (10 ng/ml, AbMole, USA) was used to stimulate RAOEC cell activation, MYLS22 (10 μM, MedChemExpress, USA) was used to inhibit OPA1 activity. The chondrogenic factor, consisting of TGF-β (10 ng/ml, Abbkine, USA), dexamethasone (100 nM), Vitamin C (50 μg/ml), proline (40 μg/ml), 1/40 ITS cell culture additive (Yuanye, China) was used to induce the chondrogenic differentiation of BMSCs.

### CCK-8 assay

A Cell Counting Kit-8 (CCK-8 kit) (Vazyme, Jiangsu) was used in accordance with the manufacturer’s regulations to evaluate the toxicity and biocompatibility of CS, ETS, and ETS/CS. Considering the disparity in concentration between CS-ONLY and ETS/CS can be attributed to the restricted drug load of ETS/CS, which could only achieve approximately 33.9%. To ensure that the total drug dosage remained relatively equivalent, the concentration of ETS/CS was approximately 3 times that of CS. RAOEC cells and BMSCs were inoculated into 96-well plates (approximately 5 × 10^3^ cells/well) and then treated with CS, ETS, and ETS/CS at the following concentrations: CS: 0, 5, 10, 25, 50, 100, 200, 500, 750, or 1,000 μg/ml; ETS/CS: 0, 30, 75, 150, 300, 600, 1,500, 2,250, or 3,000 μg/ml; ETS: 1,500 μg/ml. After 1 and 3 d, the culture medium was discarded, and 100 μl of CCK-8 detection reagent (CCK-8: DMEM = 1:10) was added to the cells and incubated for 3 h. The absorbance values of each well at 450 nm were detected using a microplate reader (Tanon, Shanghai), and the cell survival rate was calculated.

### Endocytosis of ESBP-TKSF/CTS

BMSCs and RAOEC cells were inoculated into 24-well plates (approximately 1 × 10^5^ cells/well) and FITC-ETS (1.5 mg/ml) was added. After 24 h, the cells were fixed using 4% paraformaldehyde and stained with 4′,6-diamidino-2-phenylindole (DAPI) (Yeasen, Shanghai). The adhesion and endocytosis of FITC-ETS were observed using a Leica fluorescence microscope.

### Angiogenesis capacity detection

#### Cell migration assay

Briefly, 4 × 10^4^ RAOEC cells were inoculated into the upper chamber of a 24-well Transwell insert and then supplemented with 700 μl of supplemented DMEM in the lower chamber according to the following groups: blank group (C), no intervention; activation group (T), 10 ng/ml TNF-α; SF/CS group, 10 ng/ml TNF-α and 1.5 mg/ml SF/CS; ETS/CSL group, 10 ng/ml TNF-α and 0.3 mg/ml ETS/CS; ETS/CSH group, 10 ng/ml TNF-α and 1.5 mg/ml ETS/CS; CS group, 10 ng/ml TNF-α and 0.1 mg/ml CS. After 24 h, the cells in the Transwell chamber were fixed with 4% paraformaldehyde for 10 min and stained with crystal violet (Beyotime, Shanghai) at room temperature for 15 min. Five visual fields were randomly selected for numerical analysis.

#### Tube formation assay

Matrix glue (Corning 356234, USA) was added to 48-well plates (75 μl/well) and cured at 37 °C for 1 h. Then, RAOEC cells were collected and resuspended in supplemented DMEM according to the following groups: C, T, SF/CS, ETS/CSL, ETS/CSH (the intervention concentration of each group was the same as that of cell migration experiment), and CS. A total of 4 × 10^4^ RAOEC cells were inoculated on the surface of the matrix glue. After 8 h, the cells were observed under a microscope using a 100× objective. Photos were taken from 5 random fields for observation, and angiogenesis was analyzed using ImageJ software.

### ELISA

RAOEC cells were cultured in a 6-well plate and treated with supplemented DMEM in the following groups: C, T, SF/CS, ETS/CSL, ETS/CSH, and CS (the intervention concentration of each group was the same as that of cell migration experiment). After 24 h, the cell culture supernatant was extracted, and enzyme-linked immunosorbent assay (ELISA) kits for detecting monocyte chemoattractant protein-1 (MCP-1) (SAB, USA), granulocyte-macrophage colony-stimulating factor (GM-CS) (Fcmacs, Nanjing), interleukin-8 (IL-8) (Fcmacs), and IP10 (RayBio, USA) were used to detect MCP-1, GM-CSF, IL-8, and IP10 levels, respectively, according to their respective manufacturer’s instructions.

### Mitochondrial morphology analysis

RAOEC cells were collected 24 h after treatment and fixed with 2.5% glutaraldehyde, and the intracellular mitochondrial microstructure was detected using a transmission electron microscope (TEM) (HITACHI HT7700, Japan). The cells were also collected and stained with MitoTracker Red CM-H2Xros (Yeasen, Shanghai). The distribution and content of mitochondria were determined using confocal immunofluorescence microscopy (Leica TCS SP5, Germany) and flow cytometry (Merk FlowSight, USA), respectively.

### Mitochondrial function test

RAOEC cells were collected after 24 h of treatment. The mitochondrial membrane potential of the cells was determined using a JC-1 Mitochondrial Membrane Potential Assay Kit (Yeasen, Shanghai). The activity of mitochondrial respiratory chain complex III in each group was also detected using a CheKine mitochondrial respiratory chain complex III activity detection kit (Abbkine, USA). ROS levels in cells were detected using a CheKine ROS Detection Fluorometric Assay Kit (Abbkine). Ca^2+^ levels were detected using a Ca^2+^ fluorescence probe (Yeasen).

### Coculture of RAEOCs with PMCs or BMSCs

#### Establishment of a coculture system

RAOEC cells culture supernatant was collected from each group and conditioned medium was prepared. Each conditioned medium was then cocultured with PMCs or BMSCs, while supplemented DMEM was used as a blank control. PMCs were collected after 24 h of incubation to detect changes in their migration, polarization, and secretion phenotype. The chondrogenic factors was added to the culture medium of each BMSC group, and the chondrogenic differentiation status of the BMSCs was detected 14 d later.

#### Macrophage phenotype detection

PMCs were collected from each group, inoculated into the upper chamber of a 24-well plate Transwell insert using DMEM, and 500 μl of supplemented DMEM was added to the lower chamber. After 24 h, the PMCs were fixed with 4% paraformaldehyde, followed by crystal violet staining at room temperature for 15 min. Five visual fields were randomly selected for numerical analysis. The PMCs in each group were collected and adjusted to a density of 1 × 10^6^ cells/ml and incubated with FITC-conjugated anti-rat CD11b (Proteintech, Nanjing) and APC-conjugated anti-rat CD86 antibodies (BioLegend, USA) at room temperature for 30 min. The M1 polarization level of macrophages was detected and analyzed via flow cytometry. The PMCs of each group were also collected, total RNA was extracted, and changes in the mRNA expression of inflammatory factors (TNF-α, MMP13, IL1β, iNOS) related to the macrophage secretion phenotype were detected.

#### BMSC differentiation assay

After fixation with 4% paraformaldehyde, the BMSCs in each group were stained with a toluidine blue solution (Yuanye, Shanghai) and alizarin red staining solution (Beyotime, Shanghai) to observe the chondrogenic and osteogenic differentiation of BMSCs, respectively. In addition, BMSCs were collected from each group, total RNA was extracted, and mRNA expression changes in genes related to phenotypic differentiation were detected. Toluidine blue staining and alizarin red staining were used to detect the degree of cartilage differentiation and degeneration of BMSC, respectively.

### Evaluation of cartilage regeneration in vitro

#### Construction and characterization of ETS-GM hydrogels

GelMA (EFL-GM-90, Suzhou) was purchased from Suzhou Yongqinquan Intelligent Equipment Co., Ltd. and was dissolved in 0.25% lithium phenyl to prepare a 10% (w/v) GelMA solution. ETS (1.5 mg) and 1 ml of GelMA solution were mixed evenly and solidified for 2 min in a self-made silicone mold under a blue light source with a wavelength of 405 nm to form a transparent cylindrical scaffold with a diameter of 2 mm and a length of 3 mm.

The general morphology of the ETS-GM hydrogel was observed, and its micromorphology was observed with a SEM (HITACHI Regulus 8100, Japan). The Young’s moduli of the hydrogel scaffolds were examined under axial pressure using a biomechanical tester (MTS, USA). Freeze-dried hydrogels (weight = M0) were soaked in PBS and incubated in a shaker (37 °C, 100 rpm), weighed at a predetermined time (weight = M1), and the swelling rate was calculated using the following equation:

$$\text{Swelling rate}\;\left(\text{SR}\right)\left(\%\right)\;=\;\left(M1-M0\right)/M0\;\times \;100\%$$

Similarly, freeze-dried hydrogels (weight = M0) were incubated in 5 ml of PBS containing 10 μg/ml type II collagenase in a shaker (37 °C, 100 rpm). After freeze-drying for a predetermined time, the hydrogel was weighed (weight = M1), and the degradation rate was calculated using the following equation:

$$\text{Degradation rate}\;\left(\%\right)\;=\;\left(M1-M0\right)/M0\;\times \;100\%$$

BMSCs were mixed into the ETS-GM hydrogel in a sterile environment at a density of 0.5 × 10^7^ cells/ml. After curing, 3D BMSC-containing hydrogels were cultured in 12-well plates. The adhesion status of the cells was observed under a SEM after 24 h. Calcein (Yuanye) staining and CCK-8 assays were performed on the first and third days, respectively, to evaluate the cytocompatibility of the hydrogel.

#### Coculture of RAOEC cells with PMCs or BMSCs

BMSCs were mixed into the GM hydrogel at a density of 0.5 × 10^7^ cells/ml, TGF-β (1 ng/ml) was added, then the 3D BMSC-containing hydrogels were cultured in a 6-well Transwell plate. Simultaneously, PMCs were inoculated on the upper layer. The 3D BMSC-containing hydrogels were divided into 5 groups: negative control group (N), where PMCs and BMSCs were separately cultured in supplemented DMEM; blank group (C), using the blank ROAEC culture supernatant; activation group (T), using the activated ROAEC culture supernatant; ETS/CS group, using the ROAEC culture supernatant after ETS/CS treatment; and the OPA1 inhibition group (ETS/CS-MY), using the ROAEC culture supernatant after treatment with ETS/CS and the OPA1 inhibitor MYSL22.

#### Detection of PMC polarization and BMSC chondrogenic differentiation

PMCs were collected 24 h later after treatment with the corresponding interventions, and the M1-type polarization level of macrophages was detected and analyzed using flow cytometry. In addition, the PMCs in each group were collected, and their total RNA was extracted to detect the mRNA expression changes in inflammatory factors. Two weeks later, the BMSCs were fixed with 4% paraformaldehyde and dehydrated in gradient ethanol. Then, the 3D BMSC-containing hydrogels were embedded in optimal cutting temperature compound overnight, and frozen sections were prepared using a freezing microtome. The chondrogenic differentiation of BMSCs was observed via toluidine blue staining.

### Western blot analysis

The total protein of RAOECs was extracted using a radioimmunoprecipitation assay lysis solution and quantified according to the instructions of a BCA kit (Yuanye). The SDS-PAGE Sample loading buffer was added and boiled for 10 min. The protein samples were then subjected to sodium dodecyl sulfate–polyacrylamide gel electrophoresis, electrophoretically transferred onto polyvinylidene difluoride (PVDF) membranes, and blocked with 5% milk powder at room temperature for 2 h. The PVDF membranes were incubated with antibodies against glyceraldehyde-3-phosphate dehydrogenase (GAPDH) (Proteintech), E-selectin (CD62E) (Proteintech), vascular cell adhesion molecule (VCAM) (Immunoway, USA), intercellular adhesion molecule (ICAM) (Affinity, USA), OPA1 (Proteintech), dynamin-related protein 1 (DRP1) (Proteintech), fission (FIS1) (Proteintech), P-P38 MAPK (Proteintech), P-NF-κB P65 (Affinity), P-MEK1 (Invitrogen, USA) overnight at 4 °C, and then incubated with horseradish peroxidase-conjugated goat anti-rabbit IgG (YIFEIXUE Bio Tech, Nanjing) at room temperature for 2 h. The PVDF membranes were then incubated with an ECL chemiluminescent solution (Yeasen) and protein bands were detected using a gel imaging system (Tanon, Shanghai). The gray values were measured using ImageJ software.

### Real-time quantitative polymerase chain reaction

Total RNA from RAOECs, PMCs, and BMSCs was isolated using a FastPure Cell/Tissue Total RNA Isolation Kit (Vazyme, NanJing). The isolated RNA was then reverse-transcribed into cDNA using an All-In-One 5× RT MasterMix (Abm, Canada). Real-time quantitative polymerase chain reaction (RT-qPCR) was performed using an EvaGreen 2× qPCR MasterMix (Abm). The PCR automatic serialization analyzer (ABI QuantStudio 3, USA) was set to predenaturation at 95 °C for 10 min and 40 cycles of denaturation at 95 °C for 15 s, and annealing and extension at 60 °C for 60 s. GAPDH was used as an internal reference gene. The Ct values of each target gene were obtained, and semiquantitative calculation and analysis were carried out according to the 2^−∆∆Ct^ method. The primer sequences are shown in Table.

**Table.** Target genes primer sequences used in qRT-PCR

*Hif-1α*	Forward:	5′ GTCTGAGGGGACAGGAGGAT 3′
	Reverse:	3′ CTCCTCAGGTGGCTTGTCAG 5′
*vegfa*	Forward:	5′ GCACGTTGGCTCACTTCCAG 3′
	Reverse:	3′ TGGTCGGAACCAGAATCTTTATCTC 5′
*vegfR*	Forward:	5′ GGCCACCACTCAGGACTACT 3′
	Reverse:	3′ GGCGCTTCCAAATCTCTAAC 5′
*Dll4*	Forward:	5′ TGCGGATAACCAACGACG 3′
	Reverse:	3′ GCCCACAAAGCCATAAGGAC 5′
*TGF-β F*	Forward:	5′ CAAAGACATCACACACAGTA 3′
	Reverse:	3′ GGTGTTGAGCCCTTTCCAGG 5′
*Aggrecan*	Forward:	5′ CAGTGCGATGCAGGCTGGCT 3′
	Reverse:	3′ CCTCCGGCACTCGTTGGCTG 5′
*COL10A1*	Forward:	5′ GGCAGCAGCACTATGACCCAA 3′
	Reverse:	3′ ACAGGCCTACCCAAACGTGAGTCC 5′
*COL2A1*	Forward:	5′ CTCATCCAGGGCTCCAATGAT 3′
	Reverse:	3′ TCTGTGATCGGTACTCGATGA 5′
*Sox9*	Forward:	5′ AGTACCCGCATCTGCACAAC 3′
	Reverse:	3′ ACTTGTAATCGGGGTGGTCT 5′
*Runx2*	Forward:	5′ TCACAAATCCTCCCCAAGTGG 3′
	Reverse:	3′ GAATGCGCCCTAAATCACTGA 5′
*TNF-α*	Forward:	5′ CCAGGTTCTCTTCAAGGGACAA 3′
	Reverse:	3′ GGTATGAAATGGCAAATCGGCT 5′
*MMP13*	Forward:	5′ TGGTCTTCTGATGGGCCTTC 3′
	Reverse:	3′ GTCCAGGGAGCTGCTTTTTC 5′
*IL1β*	Forward:	5′ TGTGATGTTCCCATTAGAC 3′
	Reverse:	3′ AATACCACTTGTTGGCTTA 5′
*iNOS*	Forward:	5′ CAGCATCCACGCCAAGAA 3′
	Reverse:	3′ CAGGTGTTCCCCAGGTAGGTAG
*OPA1*	Forward:	5′ GGCACTTCAAGGTCGTCTCA 3′
	Reverse:	3′ CACTGCTCTTGGGTCCGATT 5′
*Drp1*	Forward:	5′ AGGTTGCCCGTGACAAATGA 3′
	Reverse:	3′ CACAGGCATCAGCAAAGTCG 5′
*Fis1*	Forward:	5′ ACGCCTGCCGTTACTTCTTC 3′
	Reverse:	3′ GCAACCCTGCAATCCTTCAC 5′
*GAPDH*	Forward:	5′ TCGTGCGTGACATTAAGGAG 3′
	Reverse:	3′ ATGCCAGGGTACATGGTGGT 5′

### In vivo animal study

#### Animal models

All animal experiments were performed in accordance with the Guiding Principles of the Care and Use of Animals and approved by the Animal Experimental Ethics Committee of Nanjing University of Chinese Medicine (No. 202206A042). Male Sprague Dawley rats (250 ± 25 g, *n* = 75) were purchased from Jiangsu Huachuang Sino Medical Technology Co., Ltd. The rats were randomly divided into 5 groups (*n* = 15 rats in each group): sham operation group (SH), no treatment group (NT), blank scaffold control group (ETS), SF/CS scaffold group (SF/CS), and ETS/CS scaffold group (ETS/CS). The rats were anesthetized using isoflurane, and except for the SH group, and the surface of the knee trochleas of the other rats was drilled (2-mm diameter, 2-mm depth to the full layer of cartilage) [35,36]. For SH group rats, only the joint cavity was opened without drilling. The NT group was not treated, while the other groups were implanted with the 3D BMSC-containing GM hydrogels. The rats were sacrificed 12 weeks after surgery and knee samples were obtained.

#### Cartilage repair evaluation

The knee joint samples obtained were observed morphologically, and knee cartilage defects were evaluated from the aspects of filling degree, surface smoothness, regenerated tissue, texture, and integrity and scored according to the International Cartilage Repair Scoring Criteria (ICRS). The repaired knee tissue was decalcified in 19% EDTA for 14 d. The decalcified samples were then dehydrated in serial ethanol solutions, embedded in paraffin, and sectioned into 7-μm-thick sections. The sections were stained with hematoxylin and eosin (HE), toluidine blue, and type II collagen immunohistochemistry, observed under a Leica microscope, and then collagen fiber fraction (ratio of newly formed cartilage tissue area to implant pore area) was measured using a digital image analysis system (ImageJ). The new tissue structure and level of cartilage differentiation in each group were evaluated.

#### Evaluation of joint synovial inflammation

Knee synovium samples were fixed with 4% paraformaldehyde, dehydrated in serial ethanol solutions, embedded in paraffin, and sectioned into 7-μm-thick sections. The sections were stained with HE and immunohistochemical staining for MMP13 (Proteintech, Nanjing), observed under a microscope, and analyzed using ImageJ software to evaluate the level of synovial hyperplasia and inflammation.

#### Evaluation of endothelial cell activation and macrophage infiltration via immunofluorescence staining

Paraffin sections of the cartilage repair tissue and capsule were prepared. After dewaxing, gradient ethanol hydration, antigen repair by, and permeabilization, the paraffin sections were sealed with 5% bovine serum albumin at room temperature and incubated with anti-CD62E antibodies (Proteintech) overnight at 4 °C. The sections were then separately incubated with Alexa Fluor 594-conjugated goat anti-rabbit IgG H&L (Abcam, USA) and FITC-conjugated anti-rat CD11b antibodies (Proteintech) at room temperature. After using DAPI to label the nuclei, the sections were sealed with antifluorescence quenching tablets (Yuanye, Shanghai), observed under a Leica fluorescence microscope, then analyzed using a digital image analysis system (ImageJ) to assess the levels of endothelium activation and macrophage infiltration in the new cartilage and synovium.

### Statistical analysis

The experimental data are expressed as the mean ± SD. SPSS 20.0 software was used to compare the differences between groups. Statistical differences among groups were analyzed using 1-way analysis of variance (ANOVA) or 2-tailed Student test. Differences were considered statistically significant at *P* < 0.05.

## RESULTS

### Characterization of ETS

**Fig. 2.**
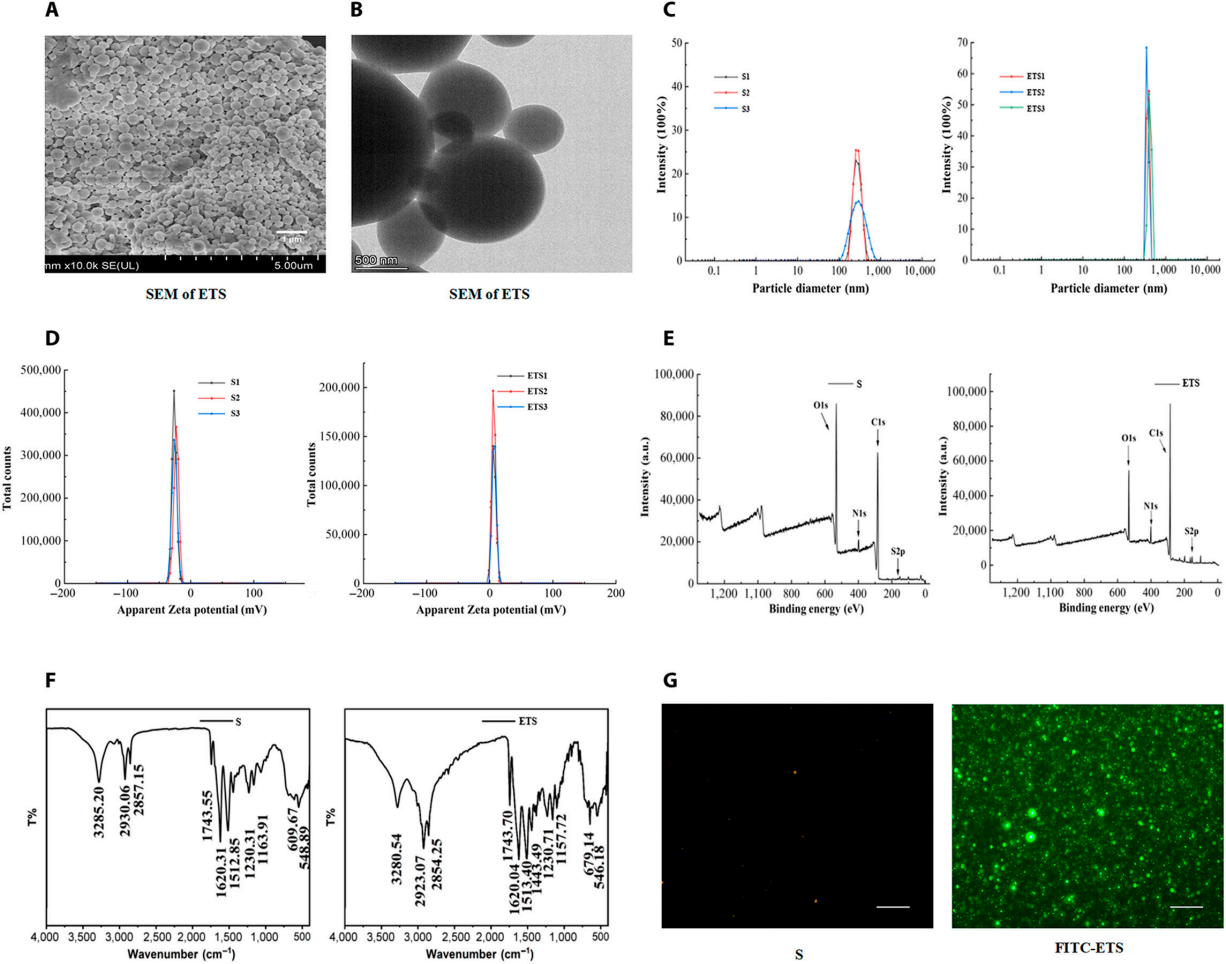
Synthesis and characterization of ETS microspheres. SEM images of ETS with different scale bars 5 μm (A) and 500 nm (B). (C) Particle size analysis of SF and ETS. (D) Apparent Zeta potential (mV) of SF and ETS. (E) XPS spectroscopy of ETS. (F) FTIR spectroscopy of ETS. (G) Fluorescent images of SF and FITC-ETS. Scale bar = 100 μm.

The SEM analysis revealed that the ETS exhibited a smooth and uniformly spherical morphology (Fig. 2A and B). As for the dynamic light scattering results, it was observed that the average zeta potential of SF was −26.1 mV, with an average size of 290.6 nm, while the average zeta potential of ETS was 5.98 mV, with an average size of 380.9933 nm (Fig. 2C and D). In terms of the FTIR findings (Fig. 2F), it was evident that the absorption peaks of ETS at wavenumbers 2,926 and 2,855 cm^−1^, associated with the stretching vibration of the -CH2- group, were markedly enhanced. Additionally, strong absorption peaks at wavenumbers 1,512 and 1,620 cm−^1^ were observed, indicating the bending vibration of the amino group. Moving on to the XPS results (Fig. 2E), ETS exhibited 2 prominent peaks at binding energies of 284.8 and 532 eV, corresponding to N 1s and S 1s respectively, which were notably intensified compared to SF. The introduction of the S element was primarily attributed to TK, while both TK and ESBP contributed to the presence of N.

Furthermore, fluorescence detection results demonstrated that FITC-ETS emitted green fluorescence at 520 nm, suggesting successful grafting of ESBP onto TK/SF microspheres through the Steglich reaction (Fig. 2G).

### Construction of the CD62E-targeting and ROS-reactive ETS/CS drug delivery system

**Fig. 3.**
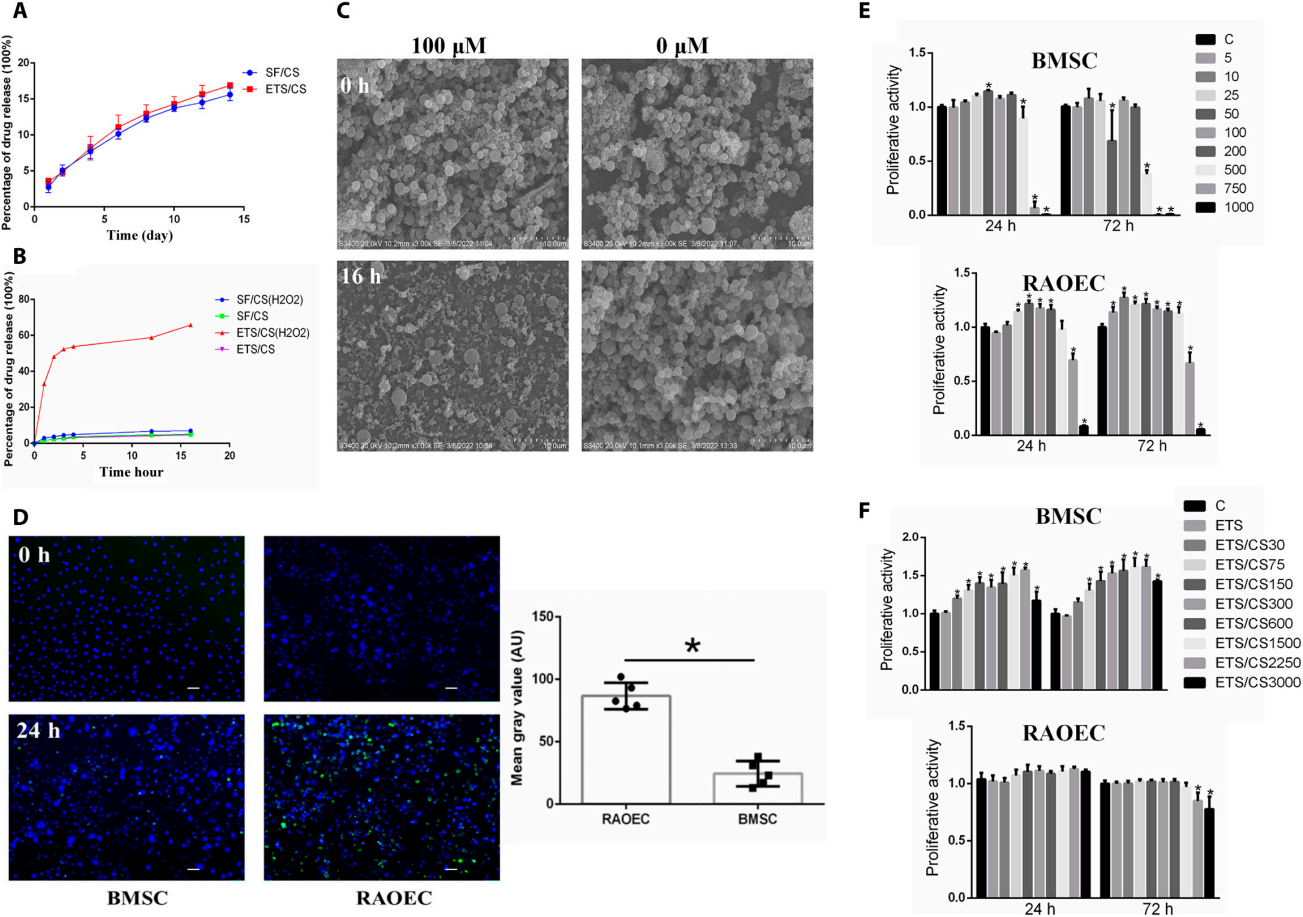
CD62E- and ROS-responsive ETS drug delivery system. H_2_O_2_ dependence release profiles of CS from ETS/CS in PBS (A) and 100 μM H_2_O_2_ solution (B). (C) TEM images of ETS/CS treated with 0 and 100 μM H_2_O_2_ solution. Scale bar = 10 μm. (D) Fluorescence images of BMSC and RAOEC cells separately incubated with FITC-labeled ETS/CS, the green fluorescence was from FITC-ETS, blue fluorescence from DAPI. *P < 0.05. Scale bar = 10 μm. Viability of BMSC and RAOEC cells after being treated with and CS (E) and ETS, ETS/CS (F) for 1 and 3 d with different concentration in vitro. Compare with C group, *P < 0.05, **P < 0.01.

The drug loading rates for ETS/CS and SF/CS were 33.9% and 37.12%, respectively. The drug release test results showed that both SF/CS and ETS/CS released CS slowly in PBS. However, in an H_2_O_2_ environment, the CS release rate of ETS/CS was substantially enhanced, reaching 60% at 16 h, whereas the release rate for SF/CS was only 3%. This indicates that the addition of TK effectively improved the ROS responsiveness of SF. SEM analysis revealed no marked degradation of SF/CS after immersion in an H_2_O_2_ solution. In contrast, ETS/CS exhibited varying degrees of degradation, with microspheres being reduced or ablated (Fig. 3A to C).

To assess the affinity of ETS toward RAOEC cells, FITC-ETS was cocultured with RAOEC cells and BMSCs. The results indicated that after 24 h of coculture, only a few microspheres were attached to or phagocytosed by BMSCs, while abundant fluorescence markers were observed on the surface of RAOEC cells. Quantitative fluorescence analysis further revealed a greater enrichment of ETS within RAOEC cells compared to BMSCs. These findings suggest that ESBP-modified ETS exhibits a targeted affinity for VECs (Fig. 3D).

The results of the CCK-8 assay indicated that ETS exhibited no significant cytotoxicity on BMSCs and RAOEC cells at 24 and 72 h, when compared to the control group. Additionally, the activity of BMSCs was significantly enhanced by CS within the concentration range of 5 to 200 μg/ml at 24 and 72 h but inhibited at concentrations exceeding 750 μg/ml. On the other hand, the proliferation of RAOECs was significantly inhibited by CS at concentrations above 500 μg/ml. Moreover, ETS/CS did not demonstrate any significant inhibitory effect on BMSCs at 24 and 72 h, when compared to the control group. Similarly, all concentrations of ETS/CS did not show any significant inhibitory effect on RAOECs at 24 h. However, at 72 h, a concentration of 1,500 μg/ml CS exhibited an inhibitory effect on RAOEC cell activity (Fig. 3E and F).

### Effect of ETS/CS on angiogenesis and endothelial cell activation in vitro

**Fig. 4.**
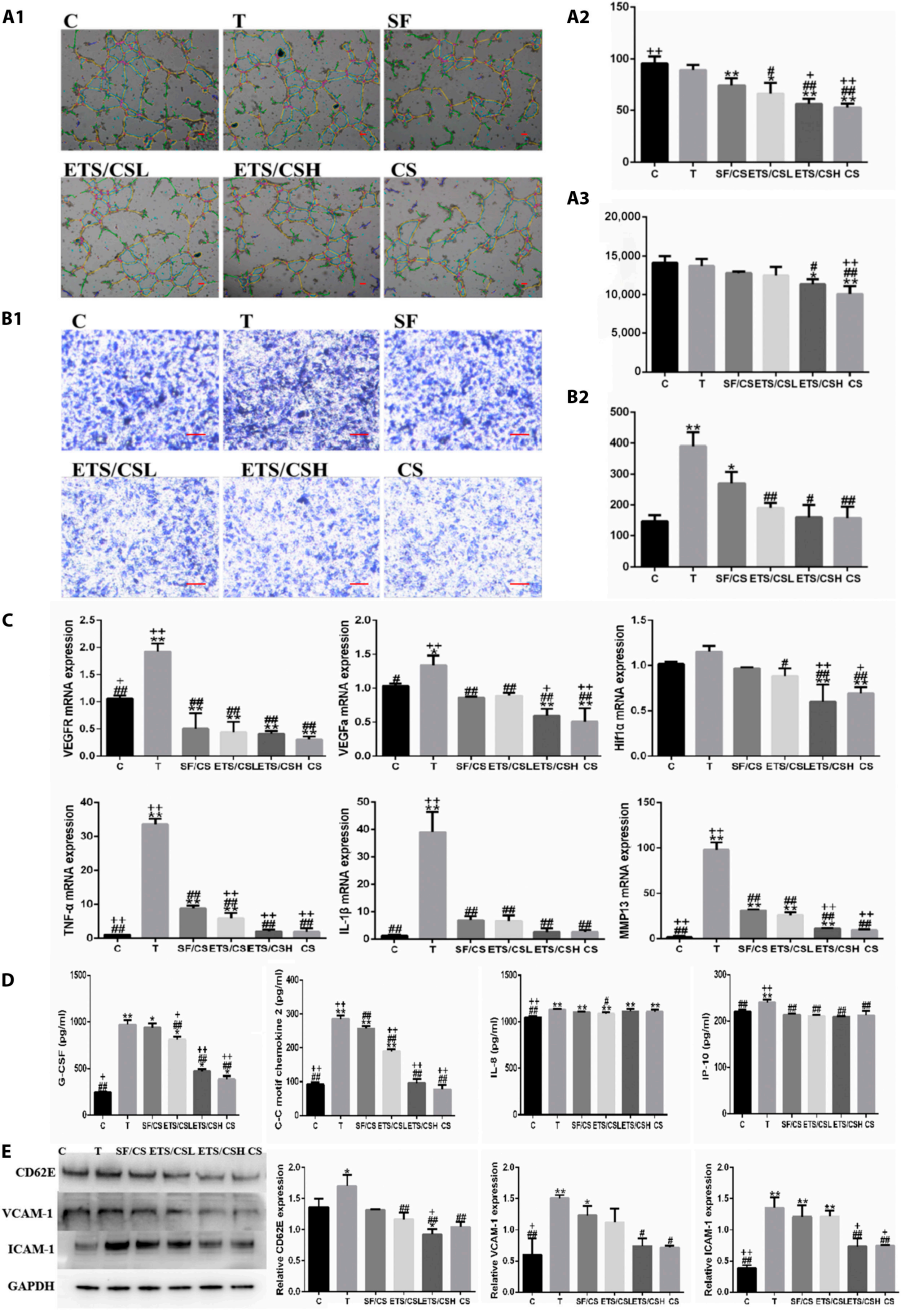
Effect of ETS/CS on angiogenesis and endothelial cell activation in vitro. RAOEC cells treated with TNF-α, and TNF-α with SF/CS (1.5 mg/ml), ETS/CSL (0.3 mg/ml), ETS/CSH (1.5 mg/ml), or CS (0.1 mg/ml) in vitro. (A1) Representative images in the tube formation assay. Quantitative analysis of branch points. Scale bar = 10 μm (A2) and cumulative tube length (A3) per microscopic field Y-coordinate. (B1) Representative images in cell migration assay. Scale bar = 100 μm. (B2) Quantitative analysis of migration cells per microscopic field Y-coordinate. (C) The mRNA expressions of 6 genes of angiogenesis and inflammation in RAOEC cells of qPCR analysis. (D) The expression of 4 chemokines in RAOEC cells of ELISA analysis. (E) Representative images and quantitative analysis of CD62E, VCAM-1 and ICAM-1 in ROAEC cell of WB analysis. Compare with C group, *P < 0.05, **P < 0.01, Compare with T group, #*P* < 0.05, ##*P* < 0.01, Compare with SF/CS group, +*P* < 0.05, ++*P* < 0.01.

The results of the angiogenesis assay (Fig. 4A) demonstrated that TNF-α stimulation led to a decrease in the length and branching of blood vessels compared to the control (C) group. Following intervention, the SF/CS, ETS/CSL, ETS/CSH, and CS groups exhibited a decreasing trend in the length and branching of blood vessels, with significant differences observed in the ETS/CSH and CS groups (*P* < 0.05) in comparison to the C and T groups. Notably, compared to the SF/CS group, both the ETS/CSH and CS groups showed stronger inhibition of angiogenesis, with ETS/CSH showing a significant difference in the number of inhibitory angiogenesis branches (*P* < 0.05).

The results of the Transwell experiment (Fig. 4B) revealed that TNF-α stimulation significantly increased the migration of RAOEC cells compared to the C group. However, after intervention, the SF/CS, ETS/CSL, ETS/CSH, and CS groups exhibited a decreasing trend in the migration of RAOEC cells, with significant differences observed in the ETS/CSL, ETS/CSH, and CS groups (*P* < 0.05) compared to the T group. Moreover, when compared to the SF/CS group, ETS/CSL, ETS/CSH, and CS showed stronger inhibition of VEC migration (*P* < 0.05).

The RT-qPCR results (Fig. 4C) demonstrated that TNF-α stimulation led to a significant increase in the mRNA expressions of VEGFA and VEGFR when compared to the C group (*P* < 0.05). However, after intervention, the SF/CS, ETS/CSL, ETS/CSH, and CS groups showed a decrease in the mRNA expressions of VEGFA, VEGFR, and HIF1α (*P* < 0.05), with the ETS/CSL, ETS/CSH, and CS groups exhibiting a more significant reduction effect (*P* < 0.05) compared to the T group. Furthermore, when compared to the SF/CS group, ETS/CSH and CS were more effective in inhibiting the mRNA expression of VEGFA and HIF1α (*P* < 0.05). Additionally, TNF-α stimulation resulted in a significant increase in the mRNA expressions of TNF-α, IL-1β, and MMP13 compared to the C group (*P* < 0.05). However, after intervention, the SF/CS, ETS/CSL, ETS/CSH, and CS groups exhibited a significant decrease in the mRNA expressions of TNF-α, IL-1β, and MMP13 in RAOEC cells compared to the T group (*P* < 0.05). Notably, when compared to the SF/CS group, ETS/CSH and CS demonstrated stronger inhibitory effects on the mRNA expressions of TNF-α and MMP13 (*P* < 0.05).

The ELISA results (Fig. 4D) indicated that TNF-α stimulation led to a significant increase in the secretion of G-CSF, CCL2, IL-8, and IP-10 compared to the C group (*P* < 0.05). However, after intervention, the SF/CS, ETS/CSL, ETS/CSH, and CS groups exhibited a decrease in the secretion of G-CSF, CCL2, IL-8, and IP-10 in RAOEC cells compared to the T group (*P* < 0.05). Furthermore, when compared to the SF/CS group, ETS/CSH and CS displayed greater efficacy in inhibiting the secretion of G-CSF and CCL2 (*P* < 0.05). The Western blot (WB) results (Fig. 4E) showed that TNF-α stimulation resulted in an increase in the expressions of CD62E, VCAM-1, and ICAM-1 in RAOEC cells compared to the C group (*P* < 0.05). Additionally, after intervention, the protein expressions of CD62E, VCAM-1, and ICAM-1 in the SF/CS, ETS/CSL, ETS/CSH, and CS groups exhibited a downward trend compared to the T group, with ETS/CSH and CS demonstrating significant effects (*P* < 0.05). Notably, when compared to the SF/CS group, ETS/CSH displayed a stronger inhibitory effect on the protein expressions of CD62E and ICAM-1 (*P* < 0.05).

### In vitro effects of ETS/CS on macrophage polarization and chondrogenic differentiation in BMSCs by regulating angiogenesis

**Fig. 5.**
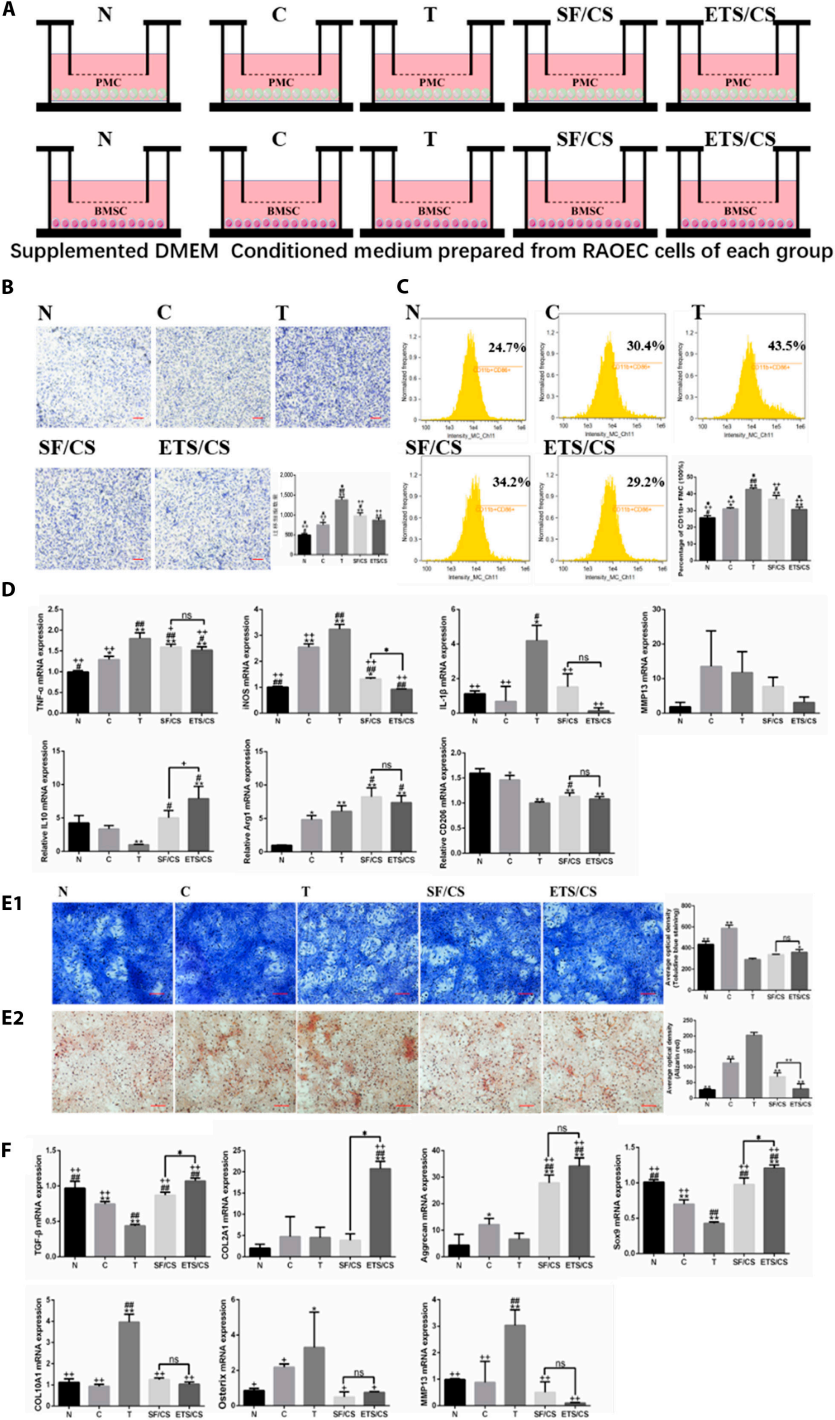
The crosstalk between VEC and Macrophage or BMSC and the effects of ETS/CS in vitro. (A). Experimental design schematic diagram showing the coculture of RAOEC cells with PMC cells or BMSC cells in a Transwell system. (B) Representative images in PMC cell migration assay. Quantitative analysis of migration PMC cells per microscopic field Y-coordinates. Scale bar = 100 μm. (C) The ratio of CD11b+CD86+ PMC cells was measured by flow cytometry. (D) The mRNA expressions of 4 genes of inflammation in PMC cells of qPCR analysis. Representative images of (E1) toluidine blue staining and (E2) alizarin red staining in BMSC cells cocultured with VEC and Quantitative analysis. Scale bar = 100 μm. (F) The mRNA expressions of 7 genes of chondrogenic differentiation in BMSC cells of qPCR analysis. Compare with N group, *P < 0.05, **P < 0.01. Compare with C group, #*P* < 0.05, ##*P* < 0.01. Compare with T group, +*P* < 0.05, ++*P* < 0.01. Compare with SF/CS group, *P < 0.05, **P < 0.01.

A Transwell assay was conducted to determine the effect of ETS/CS on the migration ability of PMCs upon coculture with RAOEC-conditioned medium (Fig. 5A). Compared with the N group, the migration rate of PMCs increased significantly after RAOEC cell supernatant stimulation (C group) (*P* < 0.05), especially after stimulation with activated RAOEC cell supernatants (T group) (*P* < 0.05). The migration rate of PMCs in the SF/CS and ETS/CS groups decreased compared with the T group (*P* < 0.05); ETS/CS inhibited PMC migration ability more strongly than the SF/CS group (*P* < 0.05). (Fig. 5B).

The polarization level of PMCs was determined using flow cytometry (Fig. 5C). Compared to the N group, the ratio of CD11b+CD86+ PMCs increased significantly especially after activated VEC supernatant stimulation (T group) (*P* < 0.05). Compared with the T group, the ratio of CD11b+CD86+ PMCs in the SF/CS and ETS/CS groups decreased significantly (*P* < 0.05). Compared with the SF/CS group, ETS/CS more strongly inhibited PMC polarization to the M1 type (*P* < 0.05). The expression of PMC inflammatory factors was detected using RT-qPCR (Fig. 5D). Compared with the N and C groups, the mRNA expressions of *TNF-α*, *iNOS*, and *IL-1β* in PMCs were significantly up-regulated after activated RAOEC supernatant stimulation (*P* < 0.05). Compared to the T group, TNF-α, iNOS, and IL-1β m RNA levels in the SF/CS and ETS/CS groups were significantly decreased (*P* < 0.05). Moreover, compared to the SF/CS group, ETS/CS significantly inhibited the expression of inflammatory cytokine mRNAs in PMCs, especially iNOS (*P* < 0.05). The expression of M2 polarization related marker genes was also detected. Compared with the N and C groups, the mRNA expressions of *IL10* and *CD206* in PMCs were significantly down-regulated after activated RAOEC supernatant stimulation (*P* < 0.05), while the mRNA expressions of Arg-1 were significantly up-regulated (*P* < 0.05). Compared to the T group, IL10 mRNA levels in the SF/CS and ETS/CS groups were significantly increased (*P* < 0.05), ARG-1 and CD206 also showed an upward trend.

Toluidine blue staining (Fig. 5E1) and alizarin red staining (Fig. 5E2) were conducted to determine the effect of ETS/CS on the chondrogenic differentiation of BMSCs upon coculture with RAOEC-conditioned medium. Compared with the N group, toluidine blue staining in the C group was stronger and alizarin red staining was weaker, especially after treatment with activated VEC supernatants. This indicates that the level of cartilage differentiation in BMSCs was reduced after treatment with activated VEC supernatants. Compared with the T group, toluidine blue staining in the SF/CS and ETS/CS groups was likewise stronger and alizarin red staining was weaker. These results showed that the level of chondrogenic differentiation of BMSC was low and could be enhanced by both SF/CS and ETS/CS.

Chondrogenic differentiation-related mRNA expression in BMSCs was also detected using RT-qPCR (Fig. 5F). Compared with the N group, *TGF-β* and *SOX9* mRNA expression was decreased in the C and T groups (*P* < 0.05). However, the expression of *COL2A1* and *Aggrecan* mRNA in BMSCs of the T group showed an upward trend, which was inhibited upon treatment with activated RAOEC cell supernatant. Compared with the T group, the TGF-β, SOX9, and COL2A1 mRNA expressions in BMSCs of the SF/CS and ETS/CS groups were significantly up-regulated (*P* < 0.05). Moreover, ETS/CS had a stronger up-regulation effect on *TGF-β*, *SOX9*, and *Aggrecan* mRNA expression than the SF/CS group (*P* < 0.01). Compared with the N and C groups, *COL10A1* and *MMP13* mRNA expression was significantly increased in the T groupB (*P* < 0.05), while the mRNA expressions of *COL10A1*, *MMP13*, and *Osterix* were significantly decreased in the SF/CS and ETS/CS groups (*P* < 0.05). Compared with the SF/CS group, ETS/CS had more strongly down-regulated *COL10A1*, *MMP13*, and *Osterix* mRNA expression (*P* < 0.05).

### Effects of ETS/CS on the mitochondrial structure during TNF-α induced VEC activation

**Fig. 6.**
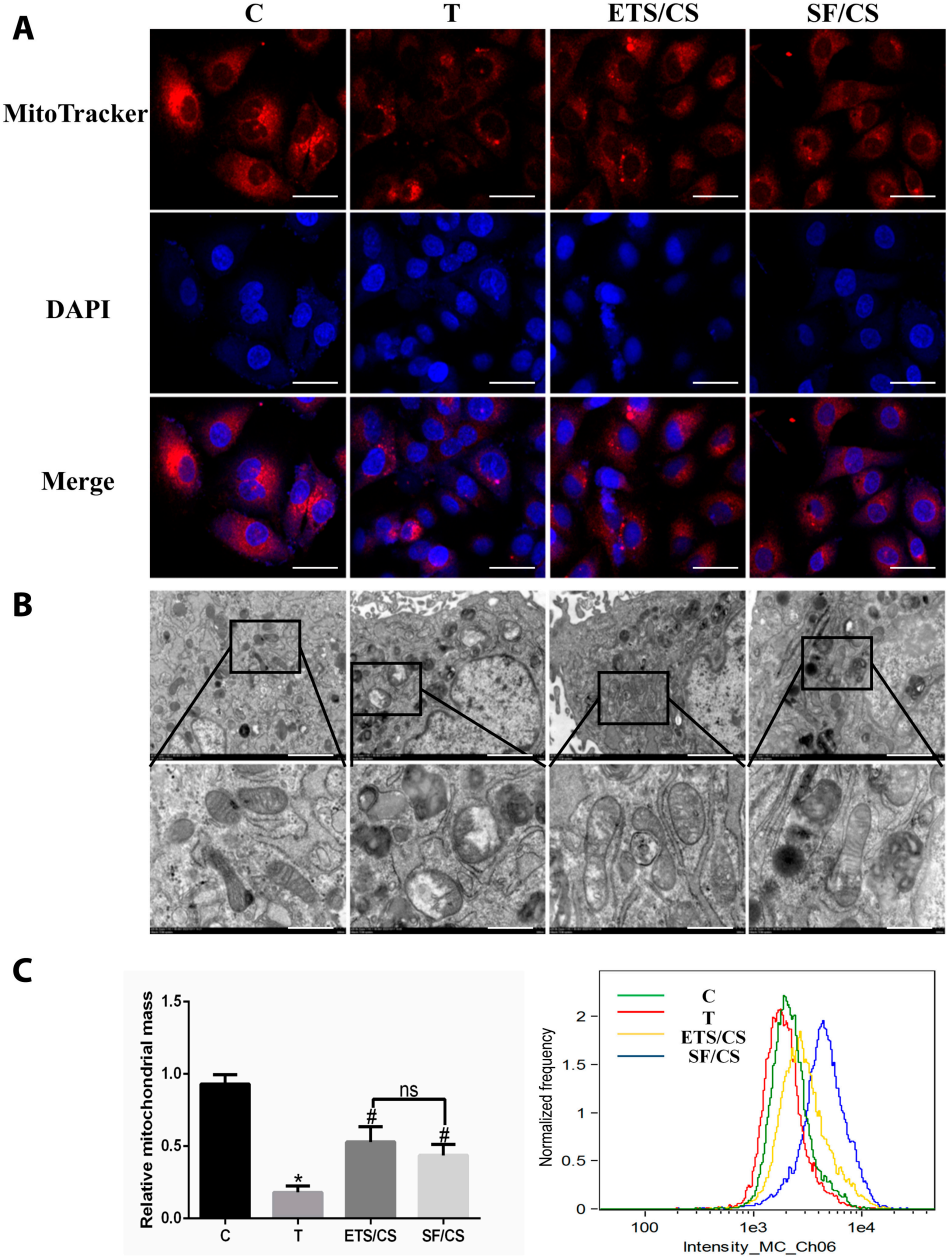
Effects of ETS/CS on mitochondrial structure during TNF-α induced VEC activation. RAOEC was activated by TNF-α stimulation and was treated with ETS/CS (1.5 mg/ml) OR SF/CS (1.5 mg/ml). (A) Representative fluorescent images in RAOEC cells mitochondrial distribution. Scale bar = 20 μm. (B) Representative TEM images of RAOEC cells mitochondrial structure. Scale bar = 1 μm and 500 nm. (C) The content of RAOEC mitochondria was detected and analyzed by flow cytometry. Compare with C group, *P < 0.05, Compare with T group, #*P* < 0.05.

To investigate the role of mitochondria in VEC activation and the influence of ETS/CS intervention on this process, the mitochondrial structure of RAOEC cells was observed (Fig. 6A and B). The results showed that the fluorescence intensity and distribution area of mitochondria decreased after TNF-α stimulation. The results of TEM showed that the inner mitochondria membrane decreased or disappeared after TNF-α stimulation. In contrast, mitochondrial fluorescence was enhanced, the mitochondrial distribution area increased, and inner mitochondrial membrane increased after treatment with ETS/CS and SF/CS. The effect of ETS/CS was slightly stronger than that of SF/CS. These results showed that the mitochondrial endometrium structure of RAOEC cells was abnormal and the endometrium ridge morphology disappeared after TNF-α stimulation; however, ETS/CS and SF/CS treatment restored the abnormal mitochondrial endometrium and ridge structure. The mitochondrial content was also detected via flow cytometry (Fig. 6C). Compared with the C group, the mitochondrial content was significantly reduced after TNF-α stimulation (*P* < 0.05). In contrast, the mitochondrial content was significantly increased after ETS/CS and SF/CS stimulation compared with the T group (*P* < 0.05). The mitochondrial content of ETS/CS-treated cells was higher than that of SF/CS-treated cells.

**Fig. 7.**
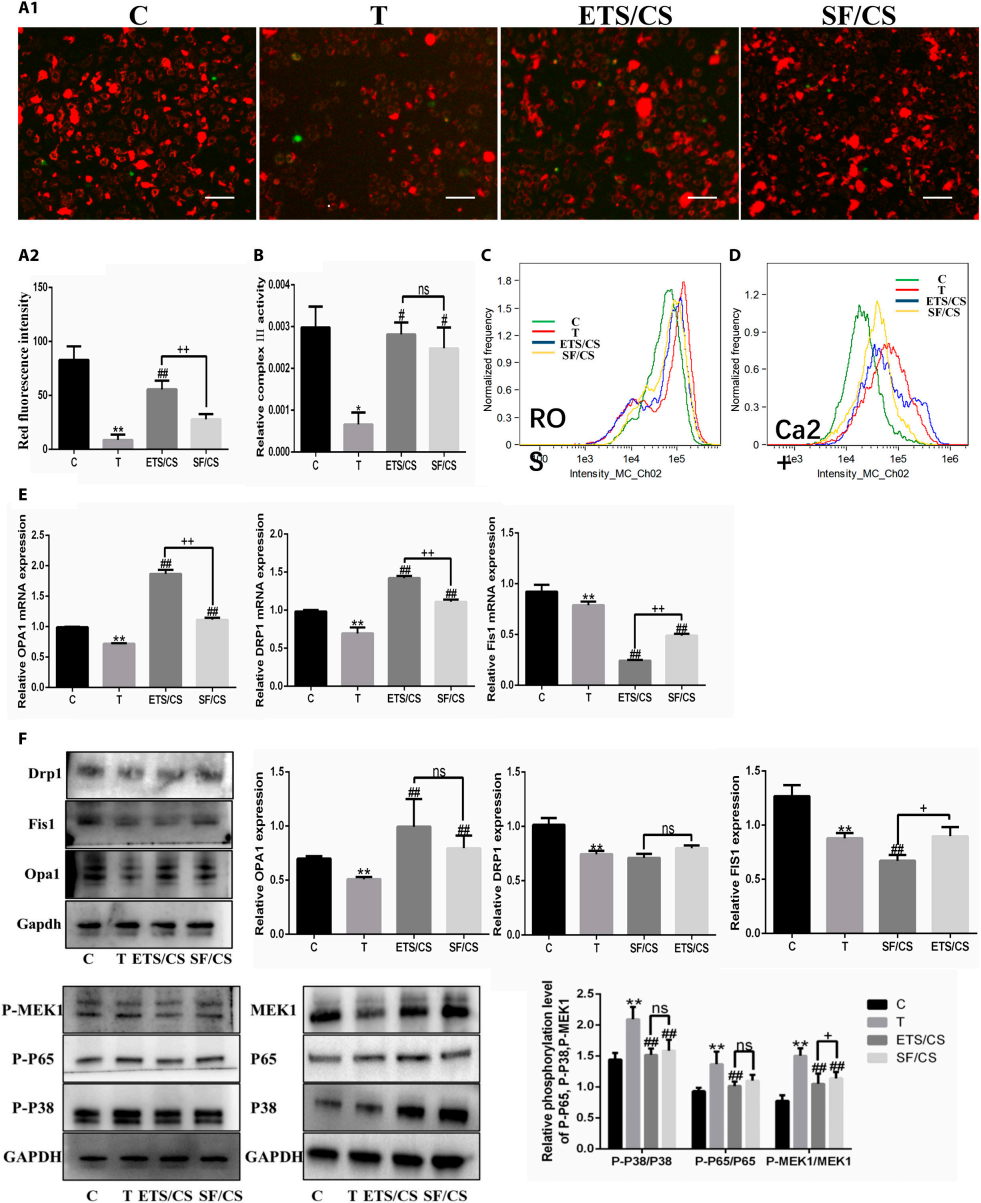
Effects of ETS/CS on mitochondrial function during TNF-α induced VEC activation. RAOEC was activated by TNF α stimulation and was treated with ETS/CS (1.5 mg/ml) and SF/CS (1.5 mg/ml). (A1) Representative fluorescent images. Scale bar = 100 μm. (A2) Quantitative analysis of RAOEC cells mitochondrial respiratory function measured by JC-1 (red fluorescence intensity). (B) Detection and Quantitative analysis of mitochondrial respiratory chain complex III activity in RAOEC cells. Intracellular ROS levels (C) and Ca^2+^ levels (D) measured by flow cytometry. (E) The mRNA expressions of 3 genes of mitochondrial fusion and fission in RAOEC cells of qPCR analysis. (F) Representative images and quantitative analysis of OPA1, Drp-1, FIS1, P-p38, P-p65, P-MEK1in ROAEC cell of WB analysis. Compare with C group, *P < 0.05, **P < 0.01. Compare with T group, #*P* < 0.05, ## *P* < 0.01. Compare with ETS/CS group, +*P* < 0.05, ++*P* < 0.01.

JC-1 staining (Fig. 7A) showed that mitochondrial respiratory function was decreased after TNF-α induction (*P* < 0.05), while it was up-regulated after ETS/CS and SF/CS intervention (*P* < 0.05). The result of the respiratory chain complex III activity test (Fig. 7B) showed that the activity of the T group was significantly decreased compared with that of the C group (*P* < 0.05), and that complex III activity was significantly up-regulated after ETS/CS and SF/CS treatment compared with the T group (*P* < 0.05). The up-regulation effect of ETS/CS on mitochondrial respiratory function and respiratory chain complex III activity was superior to SF/CS. ROS detection (Fig. 7C) assay results showed that ROS levels increased after TNF-α stimulation and then decreased after ETS/CS and SF/CS intervention, the effect of ETS/CS was stronger than that of SF/CS. Similarly, Ca^2+^ assay results also showed that Ca^2+^ levels were increased in the T group. However, compared to the T group, Ca^2+^ levels decreased in the ETS/CS and SF/CS groups (Fig. 7D).

RT-qPCR results (Fig. 7E) showed that the mRNA expressions of *OPA1*, *DRP1*, and *FIS1* in the T group were significantly decreased compared with those in the C group (*P* < 0.05). Compared with the T group, the mRNA expressions of *OPA1* and *DRP1* in the ETS/CS and SF/CS groups were significantly increased (*P* < 0.05), while that of *FIS1* was significantly decreased (*P* < 0.05). WB results (Fig. 7F) showed that compared with the C group, the protein expressions of OPA1, DRP1, and FIS1 were significantly decreased in the T group (*P* < 0.05). Moreover, compared with the T group, the expression of OPA1 was significantly up-regulated in the ETS/CS and SF/CS groups, and the effect was more significant in the ETS/CS group (*P* < 0.05). However, contrary to the RT-qPCR results, there was no significant difference in the expression of DRP1 protein between the ETS/CS and T group, whereas the expression of FIS1 decreased significantly (*P* < 0.05). Compared with the C group, the expressions of P-MEK1, P-P38 MAPK, and P-NF-κB P65 in the T group were significantly up-regulated (*P* < 0.05), while the expressions of P-MEK1, P-P38 MAPK, and P-NF-κB P65 in the ETS/CS and SF/CS groups were significantly decreased compared with the T group (*P* < 0.05), and the downward trend was more pronounced in the ETS/CS group (*P* < 0.05) (Fig. 7F).

### Effect of OPA1 on the ETS/CS-mediated inhibition of endothelial cell activation

**Fig. 8.**
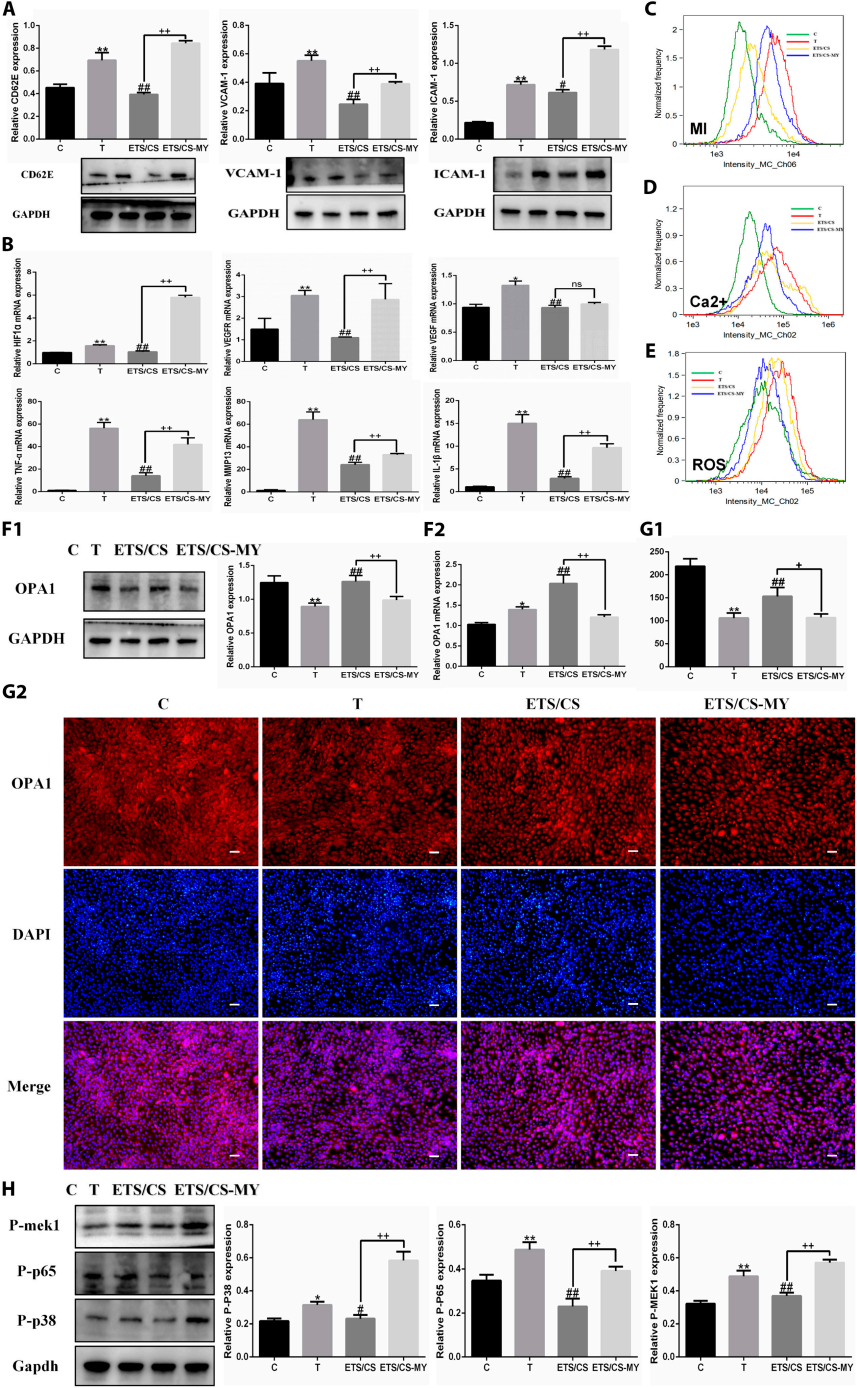
Effect of OPA1 blocking on inhibition of endothelial cell activation by ETS/CS. RAOEC was stimulated by TNF-α and interfered with ETS/CS (1.5 mg/ml), ETS/CS-MY (ETS/CS1.5 mg/ml and MYSL22 10 mM). (A) Representative images and quantitative analysis of CD62E, VCAM-1, ICAM-1 in ROAEC cell of WB analysis. (B) Proangiogenic factor and inflammatory factor mRNA expression in RAOEC cells of RT-qPCR analysis. (C) The content of RAOEC mitochondria was detected and analyzed by flow cytometry. Intracellular Ca^2+^ levels (D) and ROS levels (E) measured by flow cytometry. Protein expression (F1) and mRNA expression (F2) of OPA1 in RAOEC cells were determined by WB and RT-qPCR. Representative fluorescent images. Scale bar = 50 μm (G2) and Quantitative analysis (G1) of OPA1 (red fluorescence intensity) in RAOEC cells. (H) Representative images and quantitative analysis of P-p38, P-p65, P-MEK1 in ROAEC cell of WB analysis. Compare with C group, *P < 0.05, **P < 0.01, Compare with T group, #*P* < 0.05, ##*P* < 0.01, Compare with ETS/CS group, +*P* < 0.05, ++*P* < 0.01.

To verify the role of OPA1 in the ETS/CS-mediated inhibition of VEC activation, an OPA1 inhibitor was used. WB results showed that (Fig. 8A), compared to the C group, the protein expressions of ICAM-1, VCAM-1, and CD62E in the T group were significantly increased (*P* < 0.05). However, compared with the T group, the protein expressions of ICAM-1, VCAM-1, and CD62E in the ETS/CS group were significantly decreased (*P* < 0.05). Compared with the ETS/CS group, the expression of ICAM-1, VCAM-1, and CD62E was significantly increased after OPA1 inhibition (*P* < 0.05). RT-qPCR results showed that (Fig. 8B) compared with the C group, the mRNA expressions of *VEGFA*, *VEGFR*, *HIF1α*, and inflammatory factors such as *TNF-α*, *IL-β*, and *MMP13* in the T group were significantly increased (*P* < 0.05). In contrast, the mRNA expression of proangiogenic and inflammatory factors in the ETS/CS group significantly decreased compared with the T group (*P* < 0.05). Compared with the ETS/CS group, mRNA expressions of VEGFA, VEGFR, HIF1α, and inflammatory factors TNF-α, IL-β, and MMP13 in the ETS/CS-MY group were significantly up-regulated after OPA1 inhibition (*P* < 0.05).

Flow cytometry analysis (Fig. 8C) showed that the mitochondrial mass decreased after TNF-α stimulation and that ETS/CS could restore mitochondrial mass. However, the mass restoration effect induced by ETS/CS was weakened after OPA1 inhibition. ROS assay results (Fig. 8D) showed that the ROS levels of activated RAOEC cells were increased after TNF-α induction but decreased after ETS/CS treatment. OPA1 inhibition reversed the ETS/CS-mediated reduction of ROS levels. Ca^2+^ assay results (Fig. 8E) also showed that Ca^2+^ levels in TNF-α-activated RAOEC cells were increased, while ETS/CS treatment reduced Ca^2+^ levels. Similarly, OPA1 inhibition increased cellular Ca^2+^ levels (*P* < 0.05), reversing the ETS/CS-mediated reduction in Ca^2+^ levels.

WB, RT-qPCR, and immunofluorescence results (Fig. 8F and G) showed that OPA1 expression was significantly decreased in the T compared with the C group (*P* < 0.05). However, compared with the T group, OPA1 expression was significantly increased in the ETS/CS group, but the up-regulation effect of ETS/CS was weakened after OPA1 inhibition (*P* < 0.05). Compared with the C group, the expressions of P-MEK1, P-P38 MAPK, and P-NF-κB P65 in the T group were significantly increased (*P* < 0.05), while the expressions of P-MEK1, P-P38 MAPK, and P-NF-κB P65 were significantly decreased in the ETS/CS compared with the T group (*P* < 0.05). After OPA1 inhibition, the expression of P-MEK1, P-P38 MAPK, and P-NF-κB P6 in the ETS/CS group was significantly up-regulated (*P* < 0.05) (Fig. 8H).

### ETS/CS promotes the chondrogenic differentiation of BMSCs in vitro by inhibiting VEC activation through OPA1 up-regulation

**Fig. 9.**
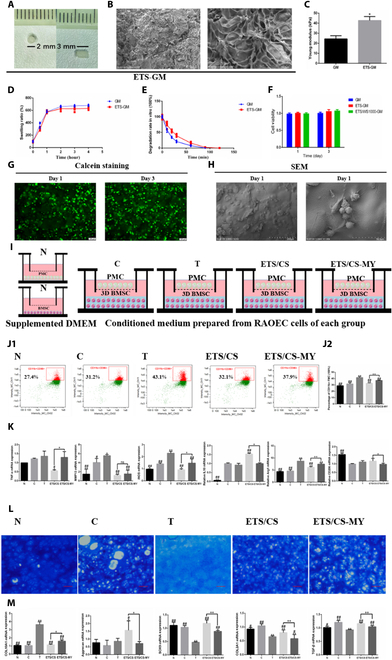
ETS/CS promoted BMSC cartilage differentiation by up-regulating OPA1 and inhibiting VEC activation. ETS-GM camera imaging (A) and the images scanning by electron microscopy (B). (C) Young's modulus of ETS-GM measured with a tensile tester. (D) Swelling properties of ETS-GM. (E) Degradation properties of ETS-GM. (F) BMSC cell activity in 3D culture with ETS-GM detected by CCK8. Fluorescence (G) and SEM images (H) in 3D-cultured BMSC with ETS-GM. (I) Experimental design schematic diagram showing the coculture of VEC with PMC and 3D BMSC in a Transwell system. (J) The proportion of CD11b+CD86+ M1 cells in coculture PMC were determined by flow cytometry. (K) PMC inflammatory factors mRNA expression in coculture PMC detected by RT-qPCR. (L) Representative toluidine blue staining images of coculture BMSC cartilage differentiation. Scale bar = 200 μm. (M) mRNA expression of coculture BMSC cartilage differentiation relative factors detected by RT-qPCR. Compare with C group, *P < 0.05, **P < 0.01, Compare with T group, #*P* < 0.05, ##*P* < 0.01, Compare with ETS/CS group, +*P* < 0.05, ++*P* < 0.01.

To simulate the process of promoting cartilage repair induced by ETS/CS in vitro, 3D BMSC cultures were performed in vitro using GM hydrogels. The constructed ETS-GM hydrogels were solidified into a nontransparent cylindrical shape with a diameter of 2 mm and a length of 3 mm (Fig. 9A). The SEM results (Fig. 9B) showed that ETS-GM has a rougher surface and denser pores than pure GM, which makes it easier for cells to adhere. The mechanical test results showed that the Young’s modulus of ETS-GM was significantly increased compared to that of GM (*P* < 0.05) (Fig. 9C). ETS-GM has good swelling properties, nearly 6 times its original mass after swelling. This property of ETS-GM can realize the adsorptive flow of nutrients, which is beneficial to the activity of BMSCs and their cartilage differentiation (Fig. 9D). ETS-GM could be degraded by type II collagenase in vitro and showed good degradability (Fig. 9E). CCK-8 assay results also showed no significant difference in the proliferation of BMSCs in the GM, ETS-GM, and ETS/CS-GM hydrogels after 1 and 3 d of culture, indicating their good biocompatibility (Fig. 9F). SEM showed that BMSCs adhered to and grew in ETS-GM, while calcein staining showed good proliferative activity in BMSCs cultured in 3D ETS-GM on days 1 and 3 (Fig. 9G and H).

A 3D coculture system of BMSCs and PMCs was also constructed, and the conditioned medium after treatment of RAOEC with TNF-α, ETS/CS, and an OPA1 inhibitor was used (Fig. 9I). Flow cytometry results (Fig. 9J) showed that, compared with the C group, the ratio of CD11b+CD86+ PMCs was significantly increased in the T group (*P* < 0.05). However, compared with the T group, the ratio of CD11b+CD86+ PMCs in the ETS/CS group decreased (*P* < 0.05), while the ratio in the ETS/CS-MY group was increased after OPA1 inhibition (*P* < 0.05), and the difference was statistically significant. The RT-qPCR results showed that (Fig. 9K), compared with the C group, the mRNA expressions of *TNF-α*, *iNOS*, and *MMP13* were significantly up-regulated in the T Group (*P* < 0.05), while the mRNA expressions of these genes were significantly decreased in the ETS/CS compared with the T group (*P* < 0.05). Compared with the ETS/CS group, the mRNA expression of *TNF-α* and *iNOS* in the ETS/CS-MY group was significantly increased after OPA1 inhibition (*P* < 0.05). At the same time, the application of MY can significantly inhibit the up-regulation of ETS/CS on IL10 and CD206 (*P* < 0.05), but the effect on ARG-1 is not obvious.

The toluidine blue staining results showed that (Fig. 9L), compared with the N group, toluidine blue staining was lighter after VEC supernatant intervention (C group), especially after activated VEC culture supernatant stimulation (T group), indicating that the cartilage differentiation of BMSCs was blocked under the dual action of activated VEC culture supernatant stimulation and PMC coculture. Compared with the T group, toluidine blue staining was more intense in the ETS/CS group, while the ETS/CS-MY group inhibited the chondrogenic differentiation effect of ETS/CS. RT-qPCR results showed that compared with the C group, the *COL10A1* mRNA level of BMSCs was significantly up-regulated in the T group (*P* < 0.05) (Fig. 9M). Moreover, compared with the T group, *COL10A1* mRNA levels in BMSCs significantly decreased after ETS/CS treatment, while *Aggrecan*, *COL2A1*, *SOX9*, and *Tgf-β* mRNA levels were significantly increased (*P* < 0.05). The mRNA expression of *Aggrecan*, *COL2A1*, *SOX9*, and *Tgf-β* was decreased, while that of *COL10A1* increased in the ETS/CS-MY group after OPA1 inhibition, which was statistically significant (*P* < 0.05).

### Effects of ETS/CS on new cartilage formation in vivo

**Fig. 10.**
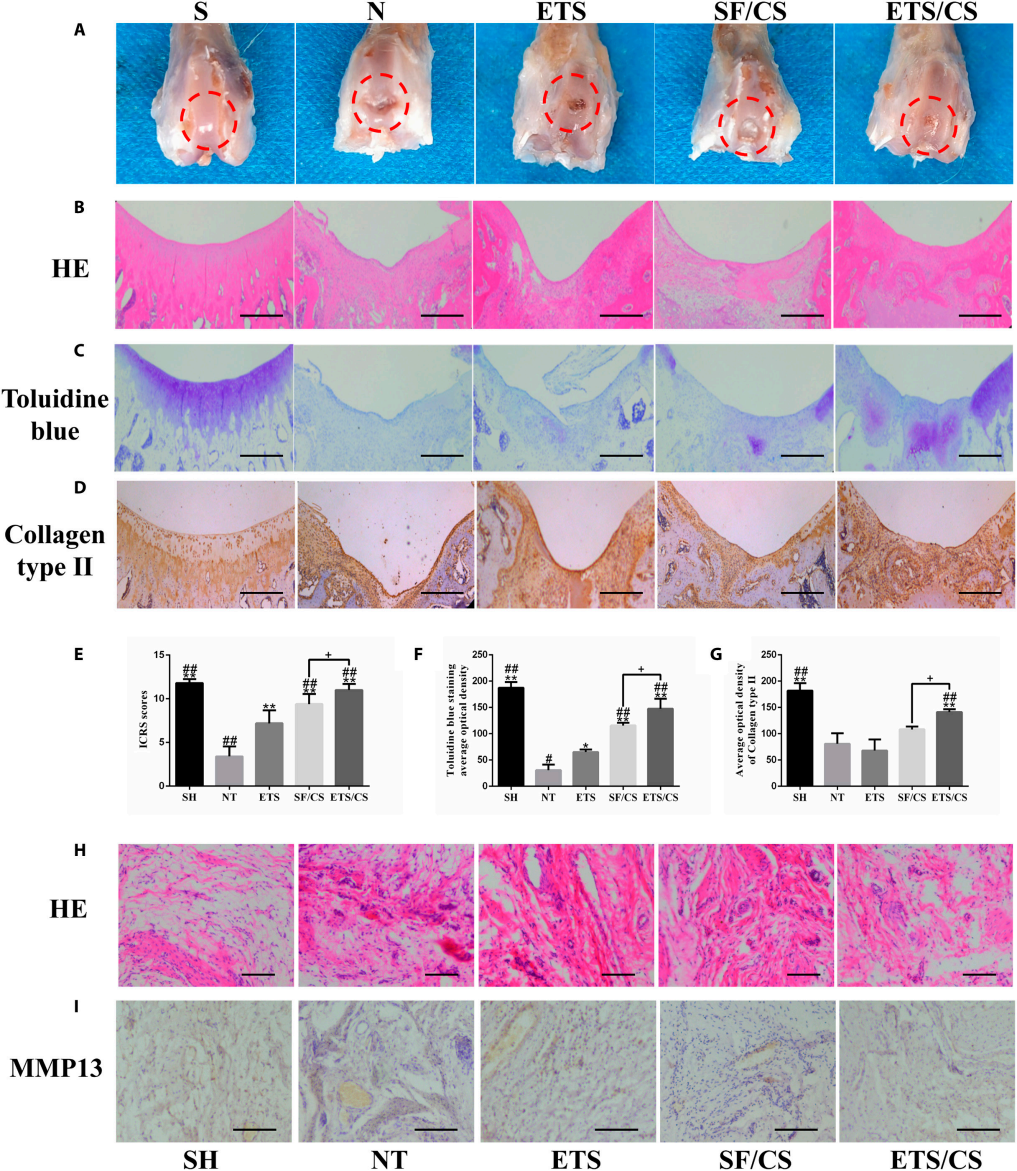
Effects of ETS/CS on endothelial activation and macrophage infiltration in neonatal cartilage in vivo. (A) Images of repairing cartilage injury in knee joint of rats. HE staining images, scale bar = 500 μm (B), toluidine blue staining images, scale bar = 500 μm (C), and collagen-II immumohistochemical staining images, scale bar = 500 μm (D). ICRS score (E), relative quantitative analysis of toluidine blue average optical density (F) and Collagen type II average optical density (G). HE staining images (H) scale bar = 100 μm and MMP13 immumohistochemical staining images, scale bar = 100 μm (I) in knee synovial of rats. Compare with NT group, *P < 0.05, **P < 0.01, Compare with ETS group, #*P* < 0.05, ##*P* < 0.01, Compare with SF/CS group, +*P* < 0.05, ++*P* < 0.01.

A rat model of knee cartilage defects was prepared to observe the effect of ETS/CS on cartilage injury repair. GM hydrogels mixed with BMSC and ETS/CS were implanted into these defects. After 12 weeks, it was observed that the articular cartilage defects of the NT group were not repaired, the cartilage defects implanted with ETS-GM were filled with tissue but not completely repaired, and the repaired surfaces were uneven (Fig. 10A). In contrast, cartilage repair in the SF/CS-GM microsphere-treated group was complete. However, the boundary between the repaired cartilage and surrounding cartilage was clear, the surface color was inconsistent with the surrounding cartilage, and the ICRS score was significantly higher than that in the NT group (*P* < 0.05) (Fig. 10E). In the ETS/CS-GM group, the articular cartilage and surrounding boundary were blurred, and the articular cartilage and surrounding articular cartilage were closely combined. The surface color and texture were the same as the normal articular cartilage, and the surface was smooth. In addition, the ICRS score of the ETS/CS-GM group was significantly higher than that of the SF/CS-GM group (*P* < 0.05).

HE (Fig. 10B), toluidine blue (Fig. 10C and F), and type II collagen immunohistochemical staining (Fig. 10D and G) were performed in the knee cartilage defects on the new tissue. The results showed that the cartilage defects in all treatment groups were filled with new tissue compared with the NT group. Toluidine blue and type II collagen staining showed increased cartilage differentiation, especially in the ETS/CS group (*P* < 0.05). Compared with the ETS group, the cartilage defects in the SF/CS and ETS/CS groups were partially or completely filled, and the surfaces were smooth. Toluidine blue and type II collagen staining showed a higher level of cartilage differentiation, especially in the ETS/CS group (*P* < 0.05). Compared with the SF/CS group, the new tissue in the cartilage defects of the ETS/CS group was fused with the surrounding primary cartilage, and the new surface cartilage was thicker. Similarly, toluidine blue and type II collagen staining showed a higher level of cartilage differentiation in this group (*P* < 0.05).

### Effects of ETS/CS on endothelial cell activation and macrophage infiltration in newly formed cartilage in vivo

**Fig. 11.**
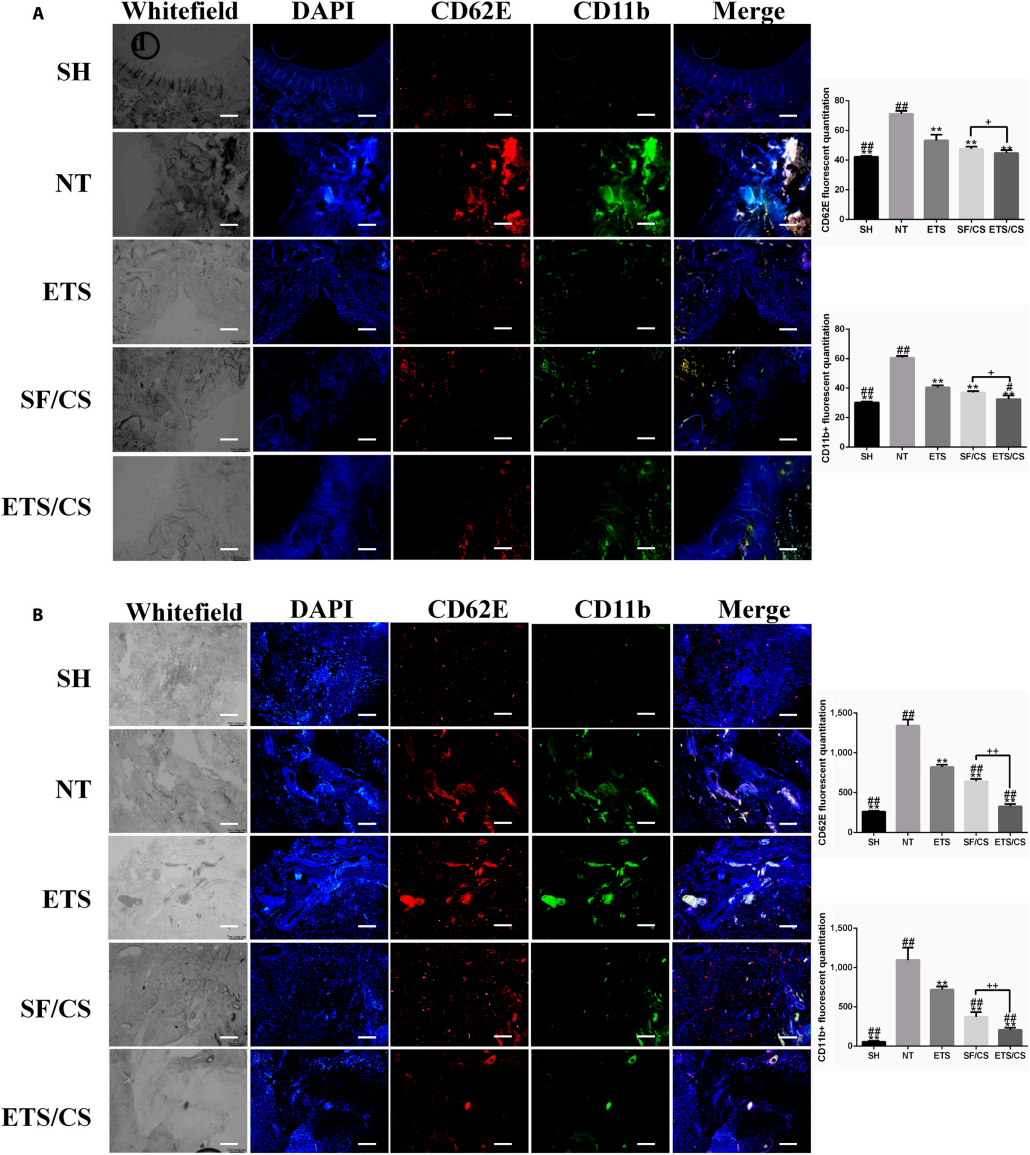
Effects of ETS/CS on endothelial activation and macrophage infiltration in neonatal cartilage and synovium of joint in vivo. The activated endothelial cells were marked with CD62E, infiltrated macrophages were marked with CD11b and the cell nucleus were marked with DAPI. (A) Representative fluorescent images and Quantitative analysis of CD62E and CD11b in neonatal cartilage. Scale bar = 200 μm. (B) Representative fluorescent images and Quantitative analysis of CD62E and CD11b in synovium of joint. Scale bar = 200 μm. Compare with NT group, *P < 0.05, **P < 0.01. Compare with ETS group, #*P* < 0.05, ##*P* < 0.01. Compare with SF/CS group, +*P* < 0.05, ++*P* < 0.01.

To observe ETS/CS-mediated VEC activation and macrophage infiltration on newborn tissue of knee cartilage defects, CD11b (macrophage marker) and CD62E (active VEC marker) were labeled and detected via immunofluorescence staining (Fig. 11A). The results showed that, compared with the SH, the fluorescence values of CD62E and CD11b in the NT group were significantly increased (*P* < 0.05), indicating that VEC activation was increased in the environment of cartilage injury, accompanied by increased infiltration of CD11B+ macrophages (*P* < 0.05). Compared with the NT, the fluorescence values of CD62E and CD11b in all treatment groups were significantly decreased (*P* < 0.05); the fluorescence values in the SF/CS and ETS/CS groups were lower than in the ETS group (*P* < 0.05). It was observed that CD62E+ cells and CD11b+ cells in the ETS group infiltrated the surface of the regenerated tissue. There were fewer CD62E+ and CD11b+ cells on the surface of the new cartilage tissue in the SF/CS and ETS/CS groups, and only a few CD62E+ cells and CD11b+ cells were found in the subcartilage, especially in the ETS/CS group. Compared with the SF/CS group, the fluorescence values of CD62E and CD11b in the ETS/CS group were significantly decreased (*P* < 0.05), indicating that the activation of VECs was decreased after ETS/CS treatment and that CD11B+ macrophage infiltration likewise decreased.

### Effects of ETS/CS on synovial inflammation, endothelial cell activation, and macrophage infiltration

To observe the effect of ETS/CS on the environment surrounding cartilage defects, HE staining and MMP13 immunohistochemical staining were performed on the synovium (Fig. 10H and I). The results showed that compared with the SH group, synovial vessel hyperplasia and inflammatory cell infiltration in the NT group were markedly increased, accompanied by increased MMP13 expression. In contrast, compared with the NT group, synovial vascular hyperplasia and inflammatory cell infiltration were decreased in each intervention group, and the expression of MMP13 was also markedly decreased. Compared with the ETS and SF/CS groups, angiogenesis and inflammatory cell infiltration and MMP13 expression markedly decreased in the ETS/CS group, indicating that synovial vascular hyperplasia and synovial inflammation in the joints were reduced after ETS/CS treatment.

CD11b and CD62E in the joint synovium were also labeled and detected via immunofluorescence staining (Fig. 11B). The results showed that, compared with the SH group, the fluorescence values of CD62E and CD11b in the NT group were significantly increased and significant angiogenesis and macrophage infiltration were observed (*P* < 0.05). These results indicate that VEC activation and the infiltration of CD11b+ macrophages increased in the environment of cartilage injury. This is consistent with the results of the VEC-PMC inflammatory cascade in vitro. Compared with the NT group, the fluorescence values of CD62E and CD11b in all treatment groups were significantly decreased (*P* < 0.05). Compared to the ETS group, fluorescence values of CD62E and CD11b in the SF/CS and ETS/CS groups were decreased (*P* < 0.05), especially in the ETS/CS group, indicating that the activation of VECs and CD11B+ macrophage infiltration decreased after ETS/CS treatment (*P* < 0.05).

## Discussion

After cartilage injury, the barrier preventing vascular invasion disappears, and new blood vessels infiltrate from the subchondral bone and synovium into the articular cartilage [37,38]. This poses challenges for tissue-engineered cartilage using stem cells. It is important to note that in the presence of local inflammation, dormant VECs become activated, migrate, and proliferate due to various stimuli. During this process, they attract leukocytes, release cytokines, and contribute to local inflammation and tissue fibrosis [39,40]. Considering the detrimental effects of VEC infiltration and activation on cartilage repair, this study employed a strategy to inhibit VECs and promote the formation of high-quality hyaluronic cartilage. To achieve this, a VEC-targeting and ROS-responsive drug delivery system based on ETS/CS was developed. This system specifically binds to CD62E, promoting VEC enrichment, and releases CS in response to excessive ROS in VECs. On one hand, it inhibits VEC activation by stabilizing mitochondrial structure and function through up-regulation of OPA1 expression. On the other hand, it down-regulates MEK1, NF-κB, and P38 MAPK signaling, thus suppressing VEC activation and the VEC-macrophage inflammatory cascade. Furthermore, this system promotes the cartilage differentiation of BMSCs and enhances the quality of repair for knee cartilage defects in rats.

Clematidis Radix, also known as WeiLingXian in Chinese, is a traditional Chinese medicine that is widely used in clinical treatment of cartilage injury due to its ability to “dispel wind and dampness and smooth meridians” [23]. CS, the main bioactive compounds in Clematis, have been found to possess anti-inflammatory properties, reduce oxidative stress, and significantly improve the repair environment of articular cartilage [41,42]. Furthermore, studies have demonstrated that these triterpenoid saponins can block abnormal microangiogenesis and effectively inhibit the activation and migration of VECs induced by inflammatory stimulation [23,43]. However, the underlying mechanism and effect of CS-mediated inhibition of VEC activation and promotion of cartilage repair is not yet fully understood.

One of the challenges associated with using CS is its unstable solubility and high molecular weight, which result in low bioavailability. To address this, we previously developed SF microcarriers loaded with CS aimed to achieve nontargeted diffusion of the drug at the site of cartilage defects after implantation. In this study, we aimed to develop an ESBP- and TK-modified SF CS-carrying system that is biocompatible and has a high drug loading rate of 339 μg/mg. In a neutral PBS environment in vitro, only a small amount of CS was released from the ETS/CS (about 15.2% for 14 d). More importantly, the ETS drug delivery system has VEC-targeted adhesion and ROS-responsive release properties. The ETS drug delivery system was developed specifically for the characterization of VECs in the context of cartilage injury. CD62E is a surface receptor that is particularly abundant in activated VECs [44]. ESBP acts as a high-affinity ligand for CD62E, facilitating the local enrichment and endocytosis of nanomedical drug carriers through binding with E-selectin. Previous research by Han et al. [29] demonstrated that ESBP-hyaluronic acid-paclitaxel micelles significantly enhanced the uptake of nanoparticles in VECs via E-selectin-mediated endocytosis. In this study, ESBP was conjugated to the SF surface through an amide bond. Fluorescence experiments confirmed the successful binding of ETS with FITC-ESBP, resulting in a significant green fluorescence signal. FTIR spectra displayed absorption peaks at 1,512 and 1,620 cm−^1^, corresponding to the amino groups. XPS analysis revealed an enhanced N absorption peak at 284.8 eV, indicating an increase in the nitrogen content introduced through the binding of ESBP to the SF surface. Notably, the ETS/CS delivery system demonstrated a stronger adhesion to VECs compared to BMSCs, offering significant advantages in achieving accurate drug delivery and increased uptake of CS by VECs.

ROS plays a crucial role as a second messenger in the regulation of cell survival and stress. Excessive ROS levels can have adverse effects on cell viability [45,46]. However, physiological levels of ROS are closely associated with the proliferation and migration of VECs in response to various angiogenesis-stimulating factors. ROS can also stimulate VEC proliferation and induce the formation of filopodia through downstream pathways such as ERK and PI3K/AKT [47–50]. Kim et al. [51] discovered that elevated ROS levels in VECs promote the formation of tip cells, their directional migration toward hypoxic regions, and the branching of blood vessels. TK is a material that responds to ROS stimuli; its mechanism involves ROS breaking the thioketone polymer chain segment and accelerating the disintegration of the microcarrier [30]. In this study, XPS results revealed a stronger S 1s peak at 532 eV in the prepared ETS compared to SF, indicating that the S content mainly originated from TK. SEM analysis demonstrated that ETS/CS microcarriers disintegrated into granules when exposed to H_2_O_2_ in vitro. The release rate of CS from ETS/CS was significantly higher than that from SF/CS, with a cumulative release rate of 60% achieved within 16 h. These results suggest that the ETS/CS system exhibits accelerated release in a ROS-rich environment.

Collectively, these findings demonstrate the successful design of the ETS/CS drug delivery system with CD62E-targeted adhesion and ROS-responsive cleavage. This system lays a solid foundation for targeted enrichment of VECs and controlled release in response to intracellular ROS cleavage in the complex environment following cartilage injury.

In our in vitro experiments, ETS/CS demonstrated superior inhibitory effects compared to SF/CS on the activation, angiogenesis, and migration of VECs induced by inflammatory stimulation. It also reduced the expression of activated VEC adhesion molecules, including CD62E. Additionally, ETS/CS down-regulated the expression of VEC angiogenesis-related genes, such as VEGFA and HIF1α, as well as inflammatory factors, which are crucial for promoting the repair of the nonvascular, noninflammatory environment in cartilage injuries.

Moreover, studies have identified the involvement of macrophages and other immune cells in the progression of articular cartilage [52–54]. ETS/CS has been found to significantly inhibit the release of VEC-related chemokines, which is beneficial for alleviating the chemotaxis of local immune cells like macrophages. In subsequent experiments, activated VECs and the conditioned medium after intervention were cocultured with BMSCs and PMCs, respectively. The results demonstrated that ETS/CS reversed the enhanced migration of macrophages, their M1-type polarization, and secretion of inflammatory cytokines induced by activated RAOEC. Additionally, it promoted the chondrogenic differentiation of BMSCs. ETS/CS also reduced the migration ability of macrophages by inhibiting VEC activation, diminishing inflammatory polarization of macrophages toward the CD11b+CD86+ M1 type, and decreasing the expression of inflammatory factors.

Activated VECs were found to adversely affect the chondrogenic differentiation of BMSCs and promote the expression of chondrofibrosis-related genes, such as COL10A1, which may be linked to the expression of provascular and proinflammatory factors in activated VECs. These results confirm that activating VECs can trigger the inflammatory cascade in macrophages and indirectly hinder cartilage repair. ETS/CS, however, can reverse this process.

Studies have revealed the close association between VEC activation and the activation of various intracellular signaling pathways, including NF-κB [55], MEK1 [15], and P38 MAPK [56] pathways. Recent research has highlighted the pivotal role of mitochondria in intracellular signal transduction during VEC activation [17,57]. Mitochondria-derived ROS and Ca^2+^ have been found to mediate the activation of NF-κB and other signals [58,59]. OPA1, which facilitates mitochondrial inner membrane fusion, regulates the mitochondrial respiratory chain complex and Ca^2+^ channels. This regulatory function is crucial in maintaining cytoplasmic ROS and Ca^2+^ levels [60–62]. In the present study, we examined the impact of ETS/CS treatment on the mitochondrial structure, function, as well as cytoplasmic ROS and Ca^2+^ levels in activated VECs. The results demonstrated that inflammatory factors stimulated and activated VECs, resulting in reduced mitochondrial inner ridge structure, decreased activity of mitochondrial complex III, elevated intracellular ROS and Ca^2+^ levels, and activation of NF-κB, MEK1, and P38 MAPK signaling pathways. However, these phenomena could be reversed by ETS/CS treatment. Additionally, when OPA1 inhibition was applied, the effects of ETS/CS on mitochondrial structure and function, ROS and Ca^2+^ levels, as well as downstream signal activation, were significantly hindered.

GelMA hydrogels were utilized for the 3D culture of BMSCs, while PMCs were cocultured with 3D BMSC-containing hydrogels using Transwell inserts. It was demonstrated that ETS/CS effectively suppressed the inflammatory differentiation of M1 macrophages by inhibiting VEC activation. Ultimately, this inhibition promoted the chondrogenic differentiation of BMSCs. However, the effects were attenuated by OPA1 inhibition. In conclusion, these findings confirm that ETS/CS inhibits VEC activation by promoting OPA1 expression, thereby maintaining mitochondrial structural and functional homeostasis, reducing ROS and Ca^2+^ levels, and activating downstream NF-κB, MEK1, and P38 MAPK signaling pathways. This cascade of events promotes the high-quality repair of cartilage defects by alleviating the VEC-macrophage inflammatory response.

To evaluate the in vivo effects, a rat knee cartilage defect model was established. GM hydrogels mixed with BMSC and ETS/CS were constructed and implanted into the defect sites. After 3 months of implantation, the ETS/CS group exhibited densely packed and well-organized hyaluronic chondroid tissue, completely fused with the surrounding tissue. In comparison to the ETS and SF/CS groups, the ETS/CS group demonstrated significant advantages in promoting cartilage defect filling and repair, as well as chondrogenic differentiation of new tissue.

Consistent with the in vitro experiments, the NT group displayed significant VEC (CD62E+) activation, accompanied by macrophage (CD11b+) infiltration in the newly formed tissues. In contrast, VEC activation and macrophage infiltration were reduced following ETS/CS treatment. The involvement of articular synovium is crucial in maintaining articular cartilage homeostasis, and synovial cell exocrine effects can negatively impact cartilage repair. Our results indicate that ETS/CS significantly inhibits synovial vascular hyperplasia, reduces the infiltration of activated VECs (CD62E+), macrophages (CD11b+), and the proliferation of synovial vessels in the synovial tissue. By reducing MMP9 expression in the synovium, the inflammatory environment resulting from articular cartilage injury can be improved, promoting the repair of cartilage defects.

In conclusion, we have successfully designed, prepared, and conducted responsiveness testing of a dual-response ETS/CS. The mechanism and superiority of the CS-loaded dual-response ETS in inhibiting TNF-α-induced VEC activation and promoting chondrogenic differentiation of BMSCs, based on OPA1-mediated mitochondrial homeostasis, were explained in vitro. Furthermore, the effectiveness of the dual-response ETS in repairing articular cartilage, by inhibiting VEC infiltration and activation, was demonstrated in vivo using a rat model of knee cartilage defects. Collectively, our findings highlight the potential of CS-loaded CD62E-targeted and ROS-responsive ETS composite hydrogels in promoting the repair of articular cartilage injuries through inhibition of VEC activation via OPA1.

## Data Availability

All data generated or analyzed during this study are included in this published article (and its supplementary information files).
